# MetaStackD A robust meta learning based deep ensemble model for prediction of sensors battery life in IoE environment

**DOI:** 10.1038/s41598-025-97720-x

**Published:** 2025-04-29

**Authors:** D. Gayathri, S. P. Shantharajah

**Affiliations:** Vellore Institute of Technology, Vellore, India

**Keywords:** Ensemble model, Meta-learning, Battery life, Sensor life, Regression model, Internet of everything, Marine life, Computational biology and bioinformatics, Environmental sciences

## Abstract

Advancements in Artificial Intelligence, Machine Learning, and Deep Learning have paved the way for ample applications in real-time. One of the major applications of this advancement is the innovative systems influenced by the Internet of Everything (IoE). The IoE environment greatly relies on the interconnection of an enormous number of sensors that are used for collecting and transmitting data. The data gathered helps for monitoring, decision-making, and automation of smart systems in multi-disciplinary domains. These sensors operate on battery power. The battery life of the sensors limits their efficiency in operation. The mechanism for analyzing the Remaining Battery Life (RBL) plays a major role in optimizing the network performance, thereby ensuring the reliability and availability of data throughout. This work focuses on proposing a novel framework integrating pre-processing, standardization, encoding scheme, and predictive modeling that includes two algorithms, RFRImpute and MetaStackD, for predicting the RBL of sensors in any IoE device using a meta-learning-based deep ensemble approach blue for analyzing factors such as power consumption, environmental conditions, operational frequency, and workload patterns. Leveraging regression algorithms such as Random Forest, Gradient Boosting, Light Gradient Boosting, Categorical Boosting and Extreme Gradient Boosting, we have modeled the non-linear and temporal dynamics of sensor battery degradation, thereby enabling proactive maintenance strategies, dynamic energy management, and resource allocation. Experimental results on the real-world Chicago Park District Beach water IoE dataset validate the effectiveness of our proposed approach, showing a 1.4% improvement in accuracy over the traditional voting ensemble model and a 93.3% reduction in training time as well as prediction time. The model size is reduced by 95.23% when compared to traditional voting ensembles.

## Introduction

The world is moving towards a fast life where people expect everything to be smart and fast. Advancements in artificial intelligence (AI) and communication technologies during the last decade have paved the way for transforming everything to become smarter and faster. The term Internet of Things (IoT)^1^ is one of the buzzwords in this fast, dynamic world where the physical world interacts with the virtual world. The interaction is achieved using various advanced technologies such as sensors, AI, Machine Learning (ML), Deep Learning (DL) and advanced communication protocols. The virtual world is developed using sensors and interconnecting these sensors to form a wireless sensor network (WSN). Each sensor communicates with other sensors and the server, where the real-time data is stored for further analysis. This setup is considered an intelligence-based information network^2^. The data collected by the sensors are transmitted to the central storage, which can be a server, base station, or cloud storage^3^. Using the data collected and shared, the AI model developed can make intelligent decisions at a critical time to make the environment smarter and safer. The term IoT^4^ is now further advanced to a paradigm called the Internet of Everything (IoE). IoE is an advancement that not only focuses on connecting just devices but also connects people, things, data and processes^5^. IoE also assists in making real-time decisions by leveraging the concepts of AI, ML, DL and Big Data analytics.

**Fig. 1:**
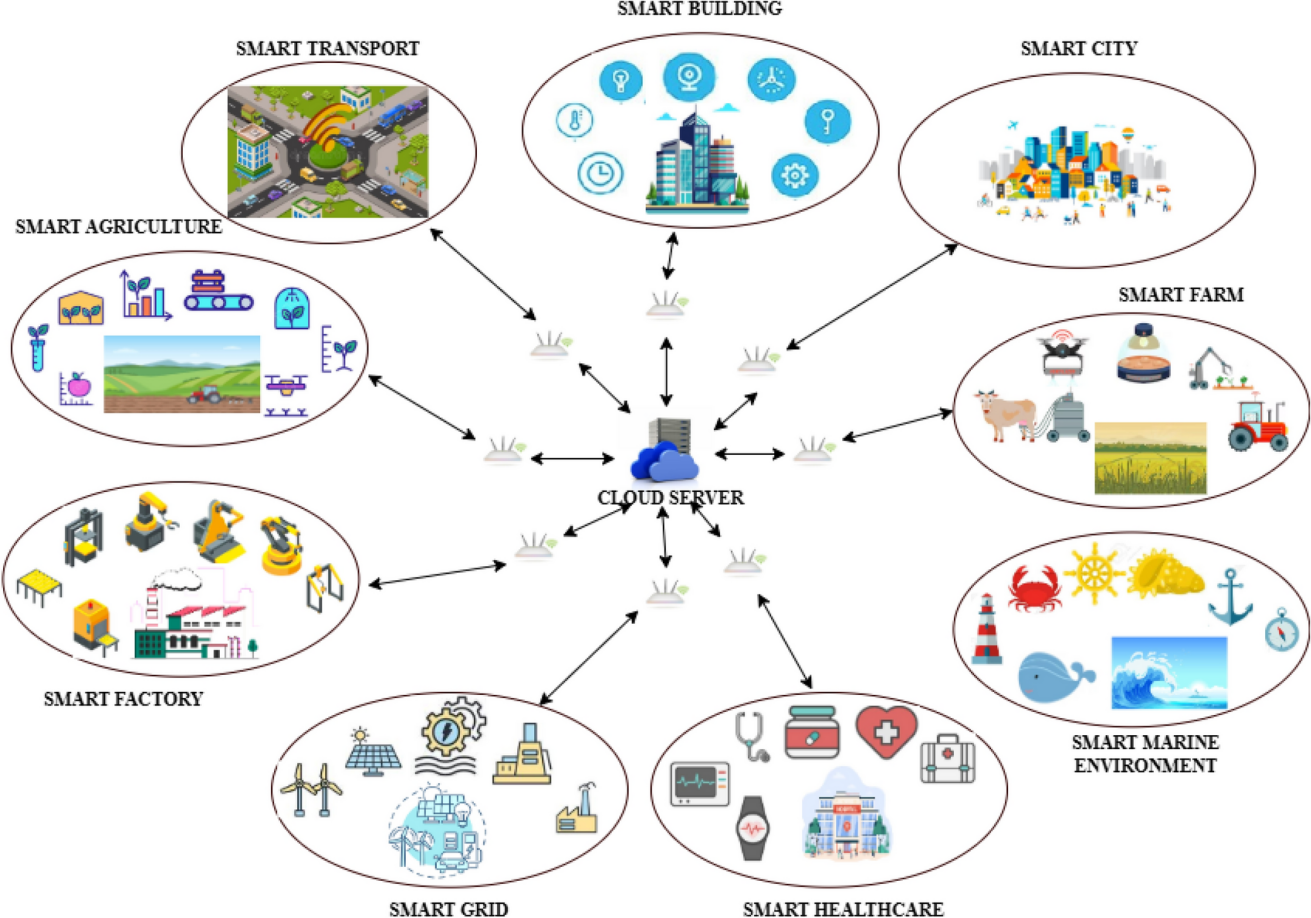
Applications of internet of everything.

From doorbells, televisions, vehicles, and refrigerators to things that can never be thought of as having intelligence, such as ice cubes and clothes, IoE has started to change the daily life of human beings around the world^6^. A few well-known smart applications of IoE are in various industrial sectors and are illustrated in Fig. 1. Few among them are smart building^7^, smart home^8^, the smart city^9^, smart transport^10^, smart industrial monitoring^11^, smart industry automation^12^, smart healthcare^13^, smart grids^14^, and smart agriculture^15^. The other industry where smart technologies can be utilized is in monitoring the marine environment^16^. This helps in protecting marine habitats as well as indicates possible disasters like tsunamis, increases in sea level, and earthquakes. Year by year, there is a huge development in our society and hence in the economy. As a result of these developments, there has been a lot of interest and focus on the marine environment recently. Currently there are multiple devices like oceanographic and hydrographic vessels that can be used for monitoring the marine environment. But these vessels are too expensive. Hence, the process of data gathering and acquisition is a challenge and not cost-effective. The process of analyzing the acquired data is time-consuming, as the data gathered is of low resolution^17^. IoE-based solutions provide very high data processing capabilities within minimal time and also add intelligence to the objects. In any marine environment monitoring system using IoT, various sensors are deployed on the seashore or in between the ocean to acquire both physical and chemical parameters. A few parameters that are usually gathered by basic IoT systems are the temperature of water, its pressure, the direction of the wind, the speed of the wind, water salinity, turbidity, pH level, availability of oxygen, the height of waves, and chlorophyll content levels. Some advanced IoT-based systems can also help in improving the physical as well as chemical parameters acquired on a timely basis. This can instantly improve the environment as well as provide safety measures.

Though there are various advantages, as discussed previously, there are also various challenges faced by the IoE environment. A few well-known challenges are security, latency, and energy optimization^18^. The major challenge that needs to be handled is energy optimization. The reason is that IoE devices are more dependent on battery-operated sensors. When sensors gather information from the real world, they need to transfer the gathered data to the nearby node so that the data can be transmitted to the base station, from where it reaches the cloud storage. Also, the nodes need to perform some basic computations before transmitting to the cloud so that immediate responses can be given by the sensor node itself during drastic conditions. For all these operations to be performed perfectly, all the sensor nodes should be alive 24 * 7 for all 365 days of the year. But in real-time this doesn’t happen as the sink nodes usually lose their energy at a very early stage, which leads to network failures though all the other sensor nodes have optimal energy to gather data. To overcome this type of failure, many researchers and academicians started to focus on developing methodologies for optimizing energy usage in sensor nodes. Some researchers focused on developing methodologies for choosing IoT sensor nodes that had optimal energy for transferring data at the end of every iteration^19^. This methodology, though it seemed to select an optimal node for data transmission, had unexpected failures. The reason for these failures was unexpected battery drainage in between. When data is not acquired, after some time the maintenance personnel would be informed to recharge the battery. But during these unexpected failures, there can be a loss of useful information, which might sometimes lead to natural disasters since predictions were lost.

A viable solution for these types of unexpected failures would be to know the remaining life of the battery in the sensor node. When the life of the battery is predicted at an early stage, the sensor life can be intimated to the maintenance personnel well in advance, hence avoiding the failure of the network. This can further help in acquiring data 24*7 without any interruption, and predictions can be made on any natural disaster. Advanced ML or DL algorithms can be utilized for predicting the remaining battery life of the sensor nodes, thereby increasing the lifetime of the IoE network for effective data acquisition. This research focuses on developing a robust, lightweight framework for predicting the remaining life of the battery in a smart marine environment using DL. The major contributions of the proposed work are as follows.

- An RFRImpute methodology is proposed to predict the missing values in the data set using the random forest regression algorithm to improve the way missing values are filled. This improves the overall performance of the final prediction model compared to the other simple techniques traditionally used to fill the missing values.
- A robust methodology is proposed for predicting the remaining life of the battery using a meta-learning-based ensemble model. This model is robust with better accuracy, less training time and improved prediction speed when compared with the traditional DL and Ensemble models.
- Comparative analysis of the proposed model with state-of-the-art methodologies and validation. This helps in providing a trusted mindset for deploying this model in a real-time IoE environment.

Due to the importance of battery life prediction, numerous researchers have worked on many machine learning and ensemble-based methods. The subsequent section gives an overview of important works in this field, and the advantages and disadvantages are pointed out.

The rest of the paper is organized as follows. Section “Related work” gives an insight into the various related research works carried out by researchers across the world in recent years. Section “Background and fundamentals” provides the fundamentals and background knowledge required for the readers to understand this research. Section “Materials and methods” describes the various methods and algorithms utilized in this study for proposed work and validation. Section “Proposed methodology” elaborates on the proposed framework for predicting the remaining life of the sensor battery. Section “Results and discussion” highlights the results obtained, performance analysis, and comparison with state-of-the-art techniques. The paper is finally concluded with the required advancements and future focus.

## Related work

The recent decade has seen many researchers who have proposed methodologies to predict the remaining life of the battery in an IoT environment. This section discusses the works related to these predictive models in recent years and summarizes their contributions as well as challenges. The researchers in^20^ conducted a study to analyze the power discharge of the battery in real time. They tried to understand the pattern of discharge, which was tried to be correlated with the battery dissipation in real-time. This was analyzed in a SPHERE sensing environment which is in a residential area. The major challenge in this work was to analyze the abnormal patterns of power discharge. Also, the pattern varied a lot among different devices, and hence, providing a general prediction model was too challenging. SPHERE is a real-time IoT platform that was developed and deployed in a residential environment for studying the behaviour of sensors and their battery usage in a real environment. Various environmental sensors were deployed in around fifty residential houses that volunteered themselves for this study^21^. The authors elaborated on the lessons they had learnt during conception as well as deployment. The major interpretation was that the remaining voltage in a battery may not be directly proportional to the remaining life of the battery. Similar to the above-discussed SPHERE platform, researchers in^22^ had developed an open, large-scale multidisciplinary architecture that can be used as a testbed by researchers worldwide. The test bed can be utilized for evaluating any research problems related to Edge Intelligence, Robotics, Smart Cities, Wireless Communication and Industrial IoT. The test bed is created using 200 multi-sensor nodes along with 20 mobile robots. Though the platform is user-friendly and widely used for research problems, the major challenge is to make it sustainable. The sustainability of this platform is primarily based on the maintenance schedule and the operational costs. The UMBRELLA platform also requires a supporting ecosystem for better adaptability based on the changing needs of the community. In^23^, the researchers proposed a novel method for the transmission and analysis of data in an IoT environment that would be energy efficient. The complete framework is divided into two phases, one focusing on the collection of data and the other on analysis. While collecting the data, the sensors lose a lot of battery backup and when this is handled, the lifetime would be better. Hence they applied a lossy compression technique on the data collected before initiation of transmission. During the second phase, the transmitted data was again rebuilt using ML techniques. Though the validation showed good results, the performance of the compressor in real-time was unpredictable. In^24^, the researchers focused on developing statistical learning mechanisms for predicting the lifetime of the battery. They claimed that simple linear models can help in providing good performance when compared with the ML and DL algorithms especially when there is not enough data set for learning. The researchers in^25^ used the random forest regression model for predicting the battery life of the sensor and validated the same using the Chicago Beach water data set. Their proposed model achieved an accuracy of 97%. The remaining useful battery life (RUL) of lithium-ion batteries was predicted by researchers in^26^ using various state-of-the-art ML algorithms. They utilized two different data sets to validate their methodology. They also introduced multi-feature multi-target feature mapping for investigating the performance of ML models in predicting the battery capacity fade and RUL in the entire life cycle.

**Table 1:** Summary of related works.

Ref.	Contributions	Data Set/Platform	Challenge
^20^	Energy consumption pattern analysis, Battery discharge pattern analysis	SPHERE platform, Residential IoT Environment	Generalized prediction model, Captured Linear patterns
^21^	IoT device design, Development experience	Bespoke sensing platform	User acceptance, Versatility
^22^	Edge intelligence analysis, Industrial IoT analysis	UMBRELLA platform	Hardware heterogeneity, Benchmarking
^23^	Novel data transmission approach, Acquired data analysis	Self-acquired sensor data	Unpredictable compression, Manual maintenance schedule
^24^	Statistical learning for prediction	IFP Graphite Cell	Time complexity, Overfitting (small dataset)
^25^	Random Forest Regression, Battery life prediction	Beach water quality sensor data	Prediction time complexity, Scalability issues
^26^	Multi-feature/target mapping, RF, XGBoost, MLP, LSTM models	Battery degradation simulation, Li-ion phosphate/graphite cells	Environment specific, Battery type dependent
^27^	Adaptive meta-model for RUL prediction	Lithium-Ion Battery Data	Uncertain Capacity Degradation, Limited High-Quality Battery Data
^28^	Quantum Particle Swarm Optimization, Extended Kalman filter	Simulated battery data	Computational Complexity of Quantum-Based Methods
^29^	Advanced LSTM for battery management	Real-world BMS Data	Inaccurate SoH and SoC Estimation, Lack of Real-Time Monitoring
^30^	Overview of RUL prediction techniques	Multiple datasets	Complex Battery Degradation, Data Availability Issues
^31^	Hybrid CNN-LSTM battery model	IoT network battery data	High Training Time for Certain Models
^32^	Feature optimization for early prediction	Experimental Battery Data	Limited Generalization, High Training Time and Memory Consumption
^33^	Domain similarity meta-learning for SOH	Spacecraft battery data	Limited Data Availability, High Computational Cost of Meta-Learning
^34^	Edge-based Deep Learning for real-time RUL	Edge device data	Computational Constraints, Limited Real-World Battery Sensor Evaluation
^35^	Federated learning for RUL prediction	Decentralized battery data	Data Privacy and Communication Overhead, Limited Generalization
^36^	ML model with meteorological variables	IoT device data	Variability in IoT Datasets, Challenges in Real-World Deployment
^37^	Personalized federated transfer learning	Heterogeneous lithium-ion battery clients	Limited Generalization to Diverse Battery Systems
^38^	Roughsets-based approach for battery life prediction	IoT data	Computational Complexity of Deep Neural Networks
^39^	Scientific reports on battery life prediction	Various datasets	Integration Challenges with IoT-Based BMS
^40^	Self-supervised learning for battery health monitoring	Unlabeled sensor data	Computational Complexity and Resource Requirements, Scalability Challenges

Recent developments in battery life prediction models have demonstrated the effectiveness of meta-learning and deep-learning methods. The research by^27^ presented a feature adaptive meta-model that enhances the prediction accuracy of lithium-ion batteries’ remaining useful life. Moreover, the research by^28^ presented a Quantum Particle Swarm Optimization Extended Kalman model, which proved effective optimization methods for prediction accuracy enhancement. Additionally,^29^ introduced an enhanced LSTM-based predictive model in the context of battery management systems, emphasizing the power of deep learning in the case of sophisticated battery degradation patterns. The paper^30^ introduced a detailed summary of different RUL prediction methods, stressing the strengths and weaknesses of different approaches.^31^ presented a hybrid deep learning architecture that blended convolutional neural networks and LSTM for precise battery life prediction with outstanding performance and adaptability improvements. The paper^32^ put forth a feature optimization technique for early battery life prediction, illustrating the benefits of improved model accuracy and less computational complexity through feature selection. In addition,^33^ proposed a domain similarity meta-learning method for lithium-ion battery state-of-health estimation in spacecraft systems, demonstrating the flexibility of meta-learning across varying operating environments. The paper^34^ proposed an edge-based deep learning framework for real-time battery life estimation with a focus on reducing latency and enhancing efficiency in edge computing scenarios. The authors in the paper^35^ utilized a federated learning mechanism for RUL prediction with an improvement in privacy preservation and model generalization, where training occurs over decentralized sources of data. The authors^36^ introduced a machine learning scheme using meteorological variables to make battery level forecasts in IoT devices, which focuses on the environmental aspect of estimating battery life. Reference 11 proposed a federated transfer learning model tailored for predicting the lithium-ion battery’s cycle life in heterogeneous clients, considering data privacy and model accuracy enhancement. This work^37^ introduced a rough sets approach to predict battery life for IoT settings based on enhanced interpretability and effective decision-making. In this work, authors^39^ provided a thorough review of battery lifetime prediction by applying different modeling approaches, offering insights into performance criteria and usability for different environments. The authors^40^ proposed self-supervised learning methods for monitoring battery health from unlabeled sensor readings, proving the ability to learn relevant insights without labeled training data, which in turn leads to more reliable battery management systems. These strategies are in agreement with the goals of MetaStackD, in addition to establishing the superiority of meta-learning in battery life prediction.

A summary of the related works focusing on contributions, limitations and challenges is tabulated in Table 1. Though many researchers focused on developing statistical, ML and DL methodologies for predicting the remaining life of the battery, these models required more computational time and resources. Moreover, most of the methodologies focused on capturing the linear relationships among the features. The other non-linear relationships, like periodicity of data acquisition, sensor idle time, and other environmental factors, are not taken into account.

Although previous research yields useful information, more understanding of ensemble learning and meta-learning is required to advance battery life prediction models. The following section explains these basic principles and how they apply to this study.

## Background and fundamentals

This section discusses in detail the various fundamental concepts required for any researcher to understand the proposed work such as Ensemble Learning, types of learning, base learners, fusion methods, Ensemble Deep Learning, the strategies followed, and the meta-learning approach.

### Ensemble learning

When any model is trained using ML algorithms, we generally land with two types of errors, one being bias and the other variance. Moreover, at the end of any model training, either we do not get promising results or the data gets over-fitted. Then, we try to rework the ML model or modify the features and continue the process until we land with promising results.

**Fig. 2:**
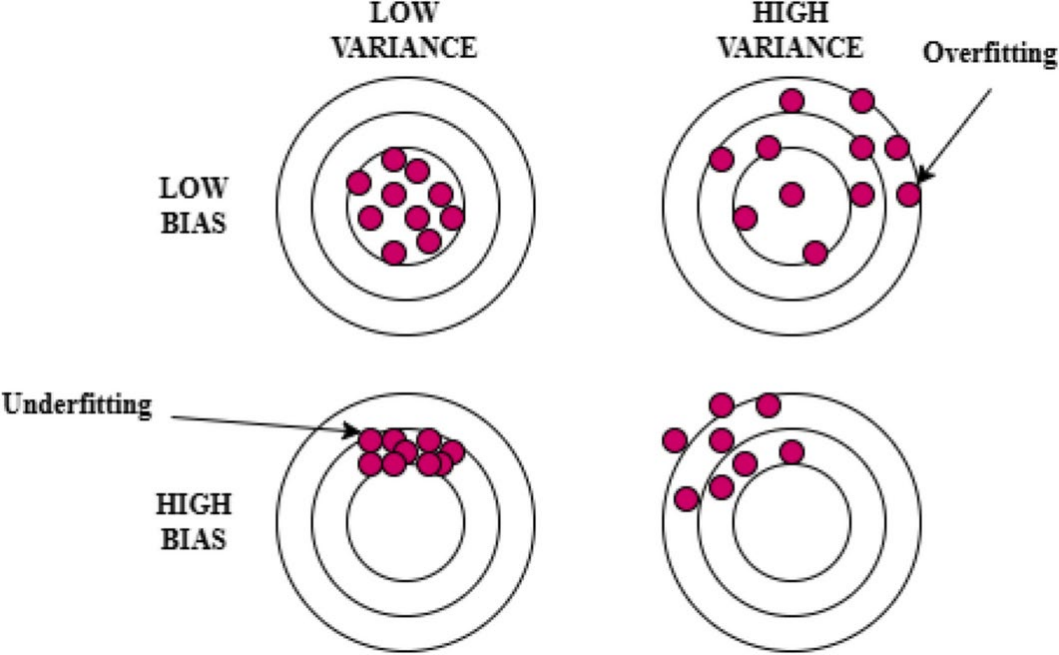
Goal of ensemble learning.

This requires more time and resources. To overcome such iterations of ML models when accuracy and robustness are critical, Ensemble Learning (EL) can be utilized^41^. EL is the process of combining various weak ML models and landing with a strong ML model. This strong ML model would provide higher accuracy. This technique reduces the time required for landing with the highly accurate and robust model when compared with the usual ML technique. When any ML model is used, the model error or the prediction error is based on three components, namely bias, variance, and noise. The prediction error is obtained by calculating the sum of bias error, variance error, and irreducible error. Bias error is calculated by finding the difference between the actual result and the result predicted by the ML model. When the difference is high, then we would say that there is high bias and this indicates that the model is under-fitting. This also specifies that the prediction model has to be modified using a stronger learner than what was used. Variance is the factor that specifies the sensitivity of the model to minor variations or alterations in the training data. When the variance is high, it means that the model is overfitting. The researcher can either increase the training data or decide on choosing a different learner, which is simpler than the current. Irreducible error is usually due to noise which can be handled by improving the pre-processing techniques. EL focuses on managing the bias and variance by using both strong and weak models in a balanced way to avoid underfitting or overfitting of the data, thereby increasing the accuracy as illustrated in Fig. 2. The various applications of EL are classification problems like spam detection and fraud detection, regression problems like stock prediction and weather forecasting, recommendation problems like movie or product recommendations, medical diagnostics, credit scoring, customer churn prediction, and portfolio optimization. The general idea behind EL is to utilize an aggregation function *E* for combining the results of *i* classifiers $L_1,$ $L_2,$ $L_3$,..., $L_i$. The output of the function *E* will be the final prediction result. Let us consider that we are given a data set D of size *x* having *j* instances and *y* features *F* represented as given in the Eq. (1).

1 $$\begin{aligned} D = \left\{ (a_j, b_j) \right\} , 1\leqslant j\leqslant x, a_j \epsilon F^y \end{aligned}$$

The EL output can be represented by Eq. (2).

2 $$\begin{aligned} b_j = \psi (a_j) = E(L_1, L_2, L3,\ldots , L_i) \end{aligned}$$

**Fig. 3:**
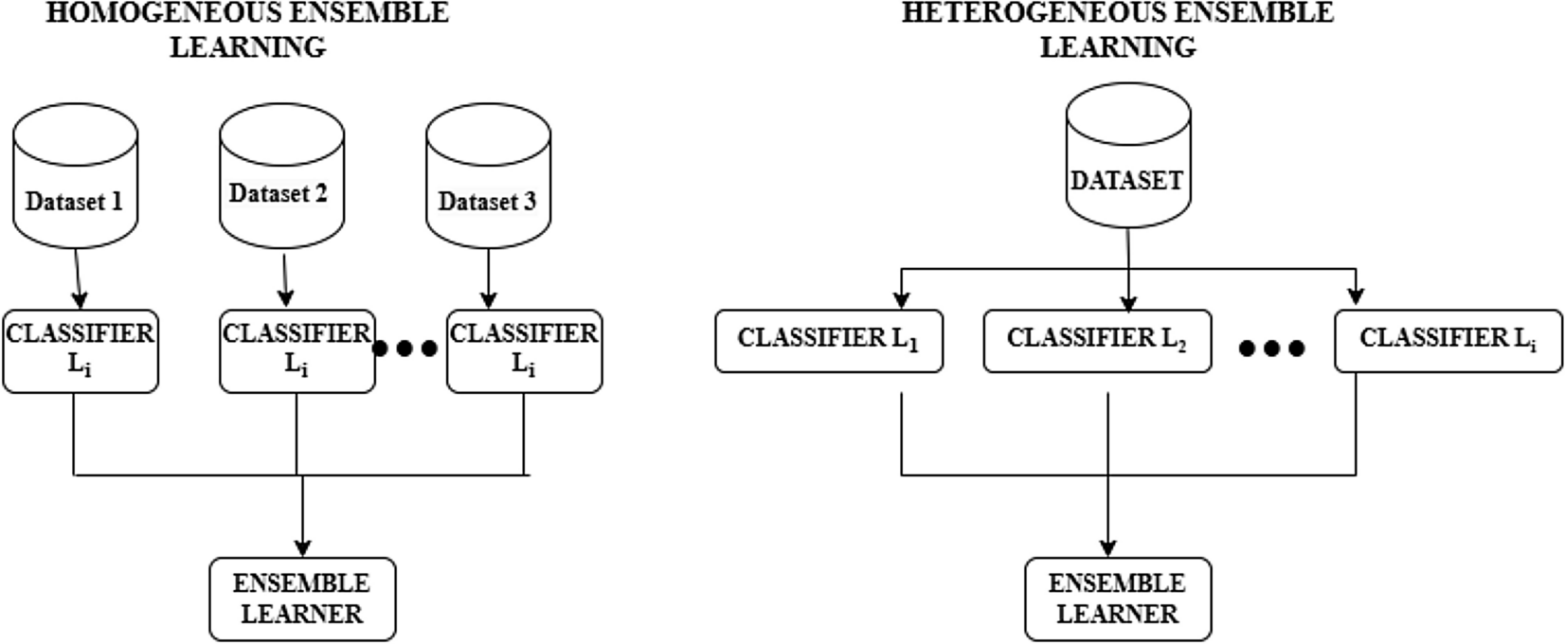
Homogeneous vs heterogeneous ensemble approach.

EL models are based on two different strategies, namely homogeneous ensembles and heterogeneous ensembles. In a homogeneous ensemble, the base classifiers used are of the same type,e and each classifier is applied on different training data to land with the final result. In the case of heterogeneous ensembles, the classifiers forming the baseline are different, and they are further applied to the same training data. The final result is obtained using any EL technique. In homogeneous ensemble methods, the feature selection method used for each classifier is the same, whereas, in heterogeneous classifiers, the feature selection methods differ from one classifier to the other. Many researchers prefer homogeneous ensemble methods as they are easier to understand and apply in real time. The selection of a suitable baseline classifier to handle the training data is a major challenge in heterogeneous ensemble methods. The basic functionality of these two ensembles can be comparatively illustrated in Fig. 3.

The performance of any Ensemble-based framework is dependent on three characteristics. The first characteristic is the type of training used in the base models, which can be sequential or parallel. The second characteristic is the type of fusion method used to combine the output of individual learners to find the final prediction result. The third characteristic is the type of Ensemble method used, either homogeneous or heterogeneous.

#### Types of base learner training

**Fig. 4:**
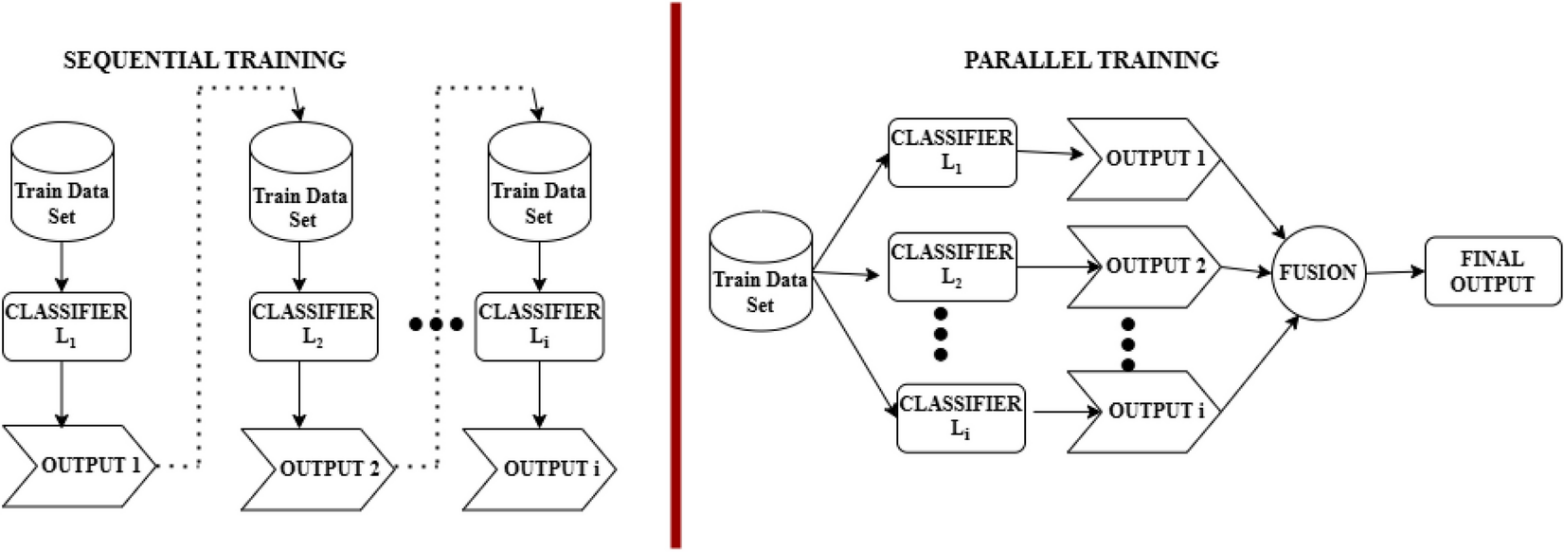
Types of base learner training.

The baseline classifiers are chosen as Ensemble members can be trained to learn individually using two different techniques, namely Sequential and Parallel Ensemble techniques^41^. In the case of the sequential ensemble technique, each learner is allowed to learn the features of the data set sequentially because there is data dependency. The errors made while learning the features using the first base model can be avoided by the second base model, thus increasing the accuracy. The major advantage of this technique is that it takes advantage of the dependence among the base learners. In the parallel ensemble technique, each base learner learns the features simultaneously since there is no dependency on the data. This technique has the advantage of exploiting the independence of the base ensemble models. In this case, the error generated by one model is independent of the other model. The final result is obtained by averaging the errors. The basic functionality of these types of training is illustrated in Fig. 4.

#### Methods of fusion

Fusion is the process of integrating the individual outputs of each classifier or learner into a single output^42^. There are two different types of fusion techniques, namely voting and meta-learning. This section describes the step-by-step procedure, the advantages, and the challenges of each technique.

##### Voting methods

Voting methods are the basic type of fusion method that can be used in almost all types of classification and regression problems for improving the accuracy of prediction. There are three types of voting methods, namely max-voting, average voting and weighted average voting.

##### Max-voting method

This was the first method used and is generally called ‘majority voting‘ or ‘hard voting. In this method, the individual class labels that are obtained as output from every base classifier are collected, and the one that has maximum votes is considered to be the final class label as in Eq. (3). There is another method in a max-voting scheme which is called as soft voting. In this scheme, the calculation is based on the probability values of the predictions made. The calculation can be made either using the maximum or average of the probability values as in Eqs. (4) and (5) where $p_1,p_2,...,p_i$ are the probability values.

3 $$b_j= mode(L_1, L_2, L_3,\ldots , L_i)$$

4 $$b_j= max\left\{ p_1(L_1), p_2(L_2), p_3(L_3),\ldots , p_i(L_i) \right\}$$

5 $$b_j= avg\left\{ p_1(L_1), p_2(L_2), p_3(L_3),\ldots , p_i(L_i) \right\}$$

This type of voting is well suited for the bagging type of ensemble technique. The major drawback of this method of max-voting fusion is its high computational cost, and it is not suitable for all types of problems.

##### Average voting method

In this method, the average of outputs obtained from each base learner is calculated, and the final class label is obtained. The average is the very basic arithmetic mean of all the probability values of the predictions made as in Eq. (6).

6 $$b_j = \frac{1}{i}\sum _{1}^{x}p_(i)$$

This method is better than the max-voting fusion method as it is more accurate and can handle the overfitting problem effectively. The drawback is that this method assumes that all classifiers chosen for prediction are effective. But in real-time, this is not the case.

##### Weighted average voting method

This is a modified version of the averaging method. In this method, each base learner is allotted a weight $z_j,$ which is directly related to the importance of the base learner model in the final result. The final result is calculated by the Eq. (7), where $d_i$ is the prediction result of the base learner. Let us consider that there are totally *n* classifiers.

7 $$\begin{aligned} d^* = \frac{\sum _{i,j=1}^{n}w_i*d_j}{\sum _{i=1}^{n}w_i} \end{aligned}$$

This method is more accurate than the average voting method, but it is computationally too expensive, and there is a challenge in fixing appropriate weights for each learner.

##### Meta-learning method

This method is usually called “Learning to Learn”. In this method, the previous learning experience is taken into account while performing the current tasks. Based on this experience, the algorithm is slightly modified or altered to improve performance. In meta-learning, there is a possibility to have more than one learning stage, and the output of each stage can be taken as input for the next learning stage, and the final output is given by the meta-learning stage. This method can be widely used in applications where the model has to adapt itself to the new data frequently. There are various meta-learning methods available out of which the most popular ones are stacking, optimization-based meta-learning methods and metric-based meta-learning methods.

**Fig. 5:**
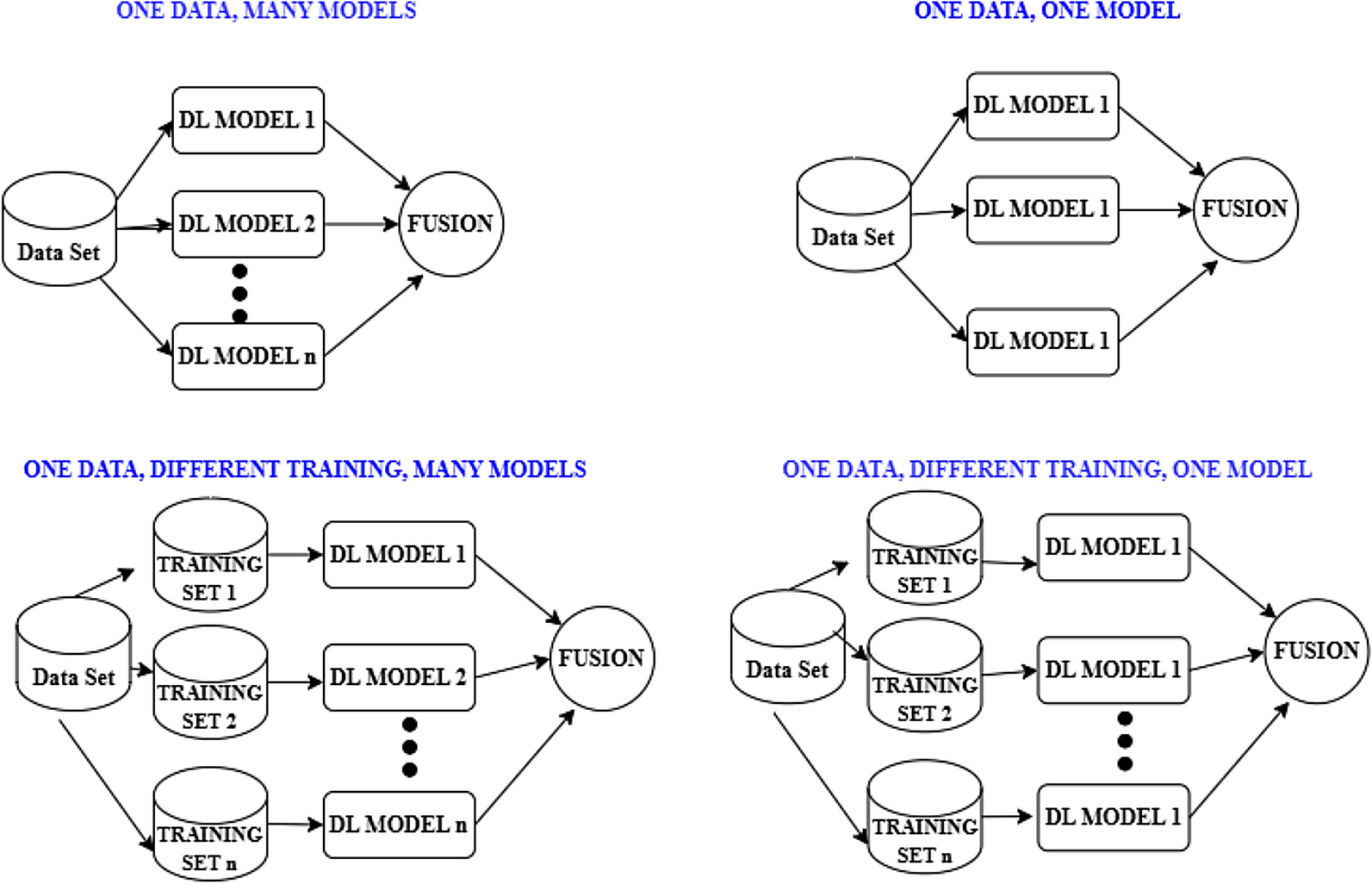
Cases of ensemble deep learning.

### Ensemble deep learning

Advancements in Deep Learning (DL) and Deep Neural Networks (DNN) during the recent decade have paved the way for vast achievements in the field of AI, such as natural language processing, computer vision, autonomous vehicles, speech recognition, etc. The DNN models are non-linear and use stochastic learning methods for learning. This implies that they can learn any relationships among the variables, though complex, and also help in approximating any mapping function for the same. However, the major challenge is that these models show a higher accuracy rate only when the variance is high. This challenge can be handled by using the ensemble technique over these models. Various models can be chosen and their results can be combined using EL to land with a final result. There are four different strategies^43^ followed, using which EL can be applied on DL models as illustrated in Fig. 5 and is listed below.

- Many different DL models can be applied to the same training data
- The Same base DL model with different structures can be used on the same training data
- Different DL models can be applied to different training data
- Same base DL model with different structures can be used on different training data

In the above-mentioned list, the first and third strategies are compatible with both DL and ML models, whereas the second and fourth strategies are compatible only with DL models. Apart from those factors already discussed in the previous section, the performance of Ensemble DL also depends on the splitting ratio of the data for training and testing. An optimal ratio among 80–20, 70–30 and 60–40 decides the performance of the ensemble system.

With a solid background in ensemble learning and meta-learning, we then explain the methodology employed to create and test our suggested MetaStackD model. Data pre-processing, model architecture, and evaluation metrics are described in the following section.

## Materials and methods

This section throws an insight into the various methods and techniques used in the proposed methodology for predicting the sensors’ battery life in an IoE environment. We highlight the ensemble base learner training technique and fusion methods, DL models used as base learners and the pre-processing methodology used for preparing the data.

### Pre-processing methodologies

Data that is acquired from the real world is always messy with noise, missing values, errors and partial information. When the acquired data has to be used by ML, DL or EL models, the data has to be of high quality to land with highly accurate results. The pre-processing is the initial step in any AI-based model, which evaluates, filters, encodes and manipulates the acquired real-time data so that its quality is increased to be used by any AI model for learning^44^. The methodologies used in this proposed work for improving its quality are discussed in this subsection.

#### Label encoding scheme

When the acquired data consists of a wide range of numerical as well as categorical data, numerical data can be handled easily. But when the categorical data is in string or label format, then these cannot be processed directly by the AI model. The label encoding scheme is the process by which the string-based categorical data is converted into numerical data. In this scheme, every category in the data set is assigned a unique integer value. The major advantage of this scheme is that it does not increase the dimension of the data set. Also, it requires less computational time and resources when compared to other encoding schemes like one-hot encoding. This scheme is best suited for ordinal sets where a sequence or order has to be maintained in the category.

#### Standard scaler method

A standard scaler is a simple method that is based on the concept of normalization. Each feature is transformed in such a way that it has a mean of ‘0’ and a standard deviation of ’1’. This transformation is done to restrict any feature from dominating the learning process due to its large magnitude. The transformation is performed using the Eq. (8) below, where $S_f$ represents the transformed value of the feature $F_i$ for $i=1,2,3,\ldots ,n$, $\mu$ is the arithmetic mean and $\sigma$ is the corresponding standard deviation.

8 $$\begin{aligned} S_f = F_i - \frac{\mu }{\sigma } \end{aligned}$$

This transformation step enhances the performance of the model, is robust towards the outliers and also helps in better interpretation ability.

### Ensemble base learners

There are various types of Ensemble learning techniques which include Bagging, Boosting, Stacking and Blending. In the case of Bagging, the training data is sampled and each sample is trained with different base learners. The final prediction is the result of the averaging of individual predictions. Boosting is a technique that follows a sequential training pattern. The error that occurred in the first model is handled by the second model. The fusion method used in this case is usually the weighted average voting scheme. In the stacking technique, the prediction obtained from the first base model is given as input for the next-level model. The prediction result obtained from the higher-level model is considered to be the final prediction result.

#### Random forest regression

**Fig. 6:**
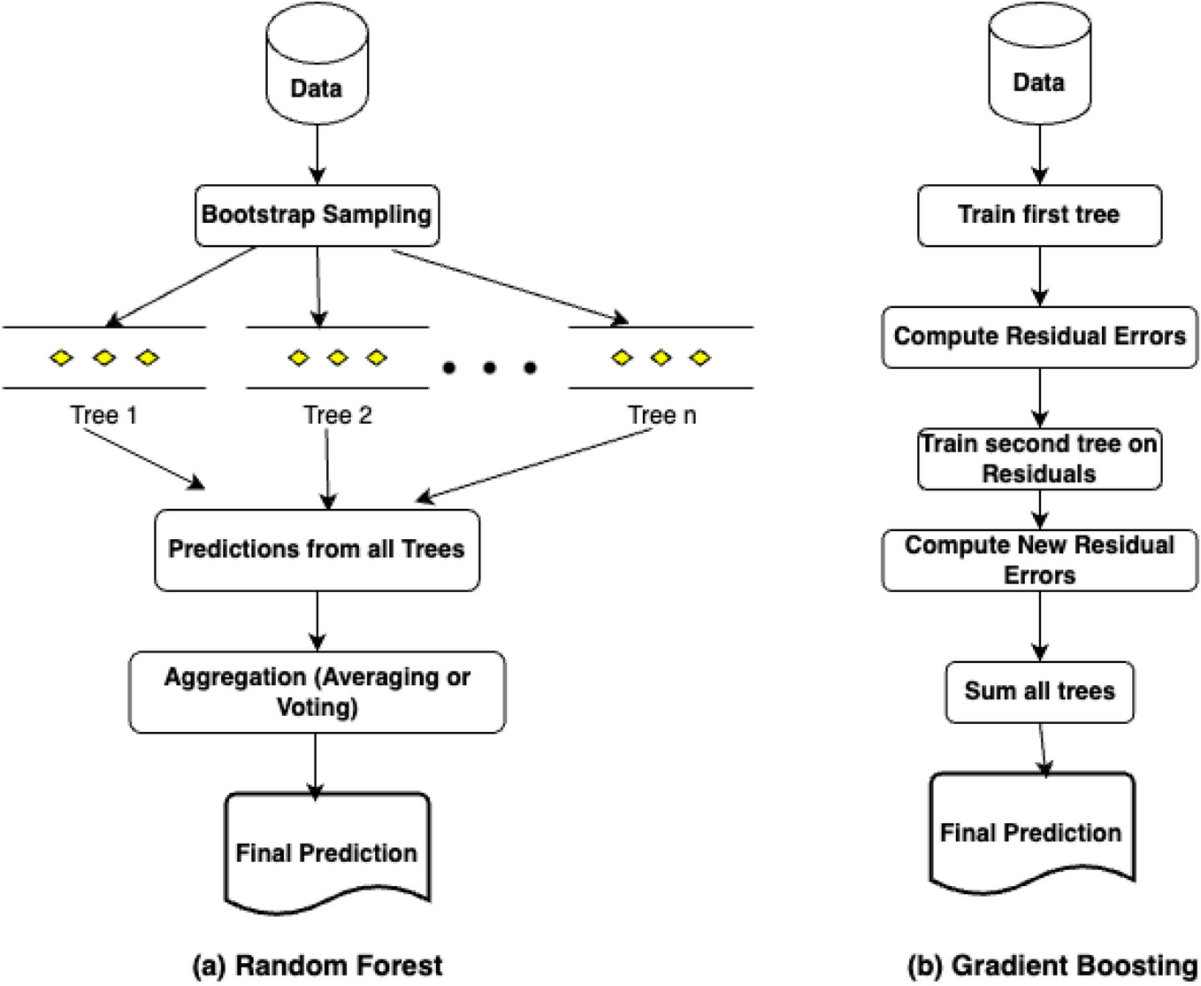
Graphical structure of random forest and gradient boosting regressor models.

Random Forest Regression^45^ is a type of Ensemble Technique that is suitable for both classification and regression problems. In this type of Ensemble, multiple decision trees are built and the prediction results are combined to produce the final prediction result using both Bootstrapping and Aggregation methods. This is generally termed as Bagging. The Random Forest model is based on multiple decision trees (DT), and each DT is considered to be a base model. Each model is trained using different samples of the data set by using row sampling and feature sampling randomly. This step is called as Bootstrapping. Here the trees built run in parallel and do not have any interaction with each other. Random subsets are selected from the training set, and small decision trees are built. These smaller DTs are then combined to form the random forest model, which gives the final result. The graphical structure of the model is illustrated in Fig. 6a below. This helps in reducing the variance and increasing the accuracy. Random Forest Regression can be used in applications where there are continuous values like stock markets, sales, etc. This model is best suited for large data sets as it can capture the non-linear relationships among the input and target variables. The major advantages of using this model are less prone to over-fitting and can perform well when we have categorical variables and high dimensions. Some important features that motivate its usage are speed, accuracy, feature selection, automation, robustness, parallelization and stability.

#### Gradient boosting

Gradient Boosting is an Ensemble technique that can be used in all types of classification and regression tasks^46^. This is a kind of Boosting technique that tries to build the best model having less prediction error by merging all the earlier models. This technique is also referred to as statistical forecasting as it tries to eliminate the prediction errors of previous models. Based on the target, Gradient Boosting can be categorized into two types, namely Gradient Boosting Regressor and Gradient Boosting Classifier. Regressor is used when the target is continuous, whereas Classifier is used when the target is a classification variable. The Loss function plays a major role in differentiating these two variants. In the case of Regressor, the loss function can be anyone out of Mean Square Error, Mean Absolute Error and Huber. In the case of Classifier, the loss function can be either Deviance Loss or Exponential Loss. If there are total *z* samples and the target variable is represented as *t*, then the Mean Square Error (MSE) can be calculated using the Eq. (9). The graphical structure of the model is illustrated in Fig. 6b.

9 $$\begin{aligned} Mean Square Error = \frac{1}{z}\sum _{i=1}^{z}(t_i - t^*_i) \end{aligned}$$

where $t_i$ is the actual target and $t^*_i$ is the predicted target. Similarly, the Mean Absolute Error can be given by Eq. (10).

10 $$\begin{aligned} Mean Absolute Error = \frac{1}{z}\sum _{i=1}^{z}\left| t_i - t^*_i \right| \end{aligned}$$

The Deviance loss, which can also be referred to as Log loss or binary cross entropy loss, can be calculated using the Eq. (11). Here, $p_i$ is the probability of prediction for Class $C_n$ where *n* specifies the number of class labels in the classification problem.

11 $$\begin{aligned} Log Loss = -\frac{1}{z}\sum _{i=1, j=1}^{i=z, j=n}\left[ t_i log(p^*_i(C_n)) + (1-t_i)log(1-p^*_i(C_n)) \right] \end{aligned}$$

The Exponential Loss can be used in Boosting techniques like AdaBoost and can be represented as given in Eq. (12).

12 $$\begin{aligned} Exponential Loss = \frac{1}{z}\sum _{i=1}^{z}\exp (t_i - t^*_i) \end{aligned}$$

#### Categorical boosting

**Fig. 7:**
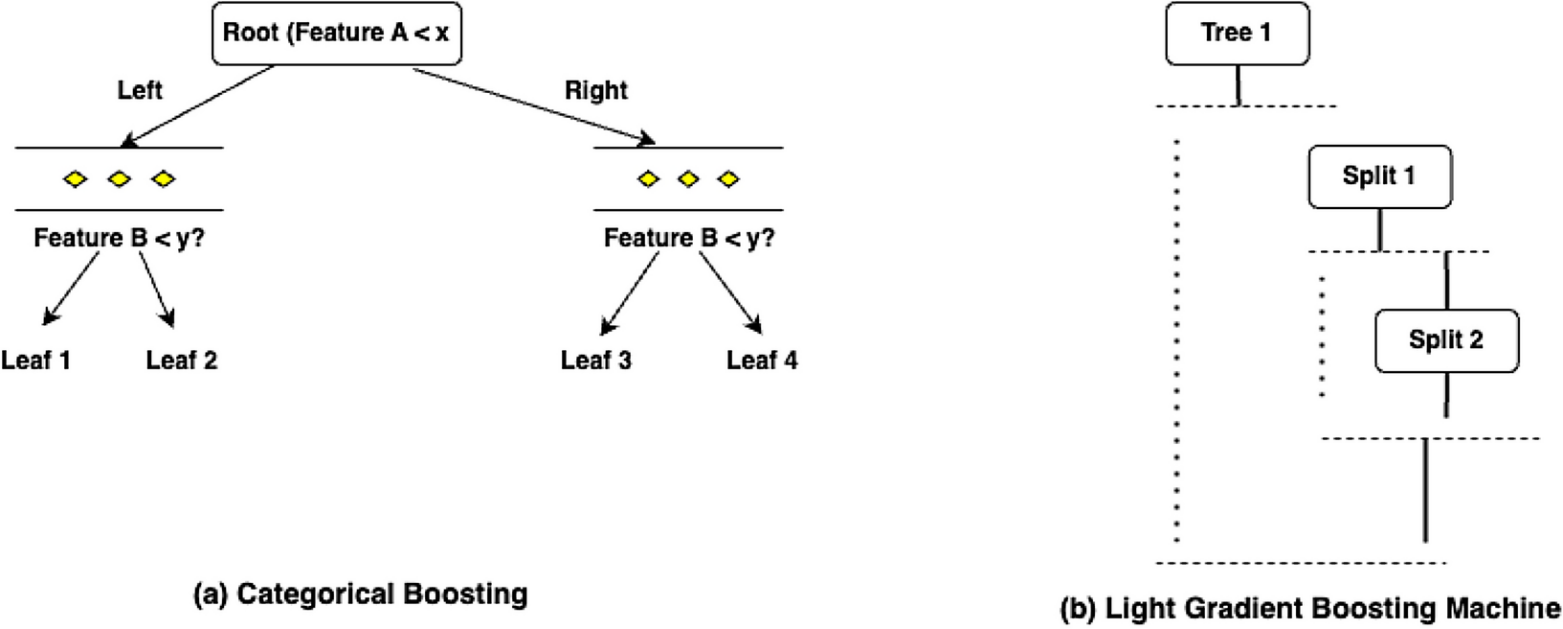
Graphical structure of CatBoost and Light GBM models.

Categorical Boosting (CATBoost) is a type of Gradient Boosting mechanism that was developed by Yandex. This method can efficiently handle classification, regression and ranking tasks. They are best suited for categorical features. It is well known for its high speed and accuracy. The CatBoost algorithm functions using the oblivious trees instead of normal decision trees. In the case of oblivious trees, each node available at the same level uses similar splitting conditions concerning feature and threshold, as shown in the Fig. 7a. Also, unlike the other gradient boosting methods like Extreme Gradient Boosting and Light Gradient Boosting, this method can encode the categorical features automatically using ordered boosting and permutation-based encoding. Since ordered boosting is used for calculating the leaf values, over-fitting issues are handled. This also helps in preventing the target leakages. The method used for encoding is Categorical Target Statistics (CTR). Let us consider that the target categorical feature to be encoded is $F^i_c,$ and the encoding is done using the Eq. (13) below.

13 $$\begin{aligned} CTR(F^i_c) = \frac{\sum _{j=1}^{n}y_j.I(F^j_c = F^i_c)}{\sum _{j=1}^{n}I(F^j_c = F^i_c)} \end{aligned}$$

where $I(F^j_c = F^i_c)$ is an Indicator function whose value can be either 1 or 0 based on True or False values, respectively. In the fraction shown in Eq. (13), the numerator is the summation of the target values for which the indicator I is 1. The denominator represents the total number of such target occurrences. After this initial pre-processing step, the oblivious trees are constructed. Then, the gradient boosting process is carried out. The initial step here is to calculate the residuals using the Eq. (14) given below.

14 $$\begin{aligned} r_i = y - P_{i-1}(x) \end{aligned}$$

where $r_i$ is the residual error at the *i*th iteration, *y* is the actual value and $P_{i-1}(x)$ is the value predicted in the previous iteration. The model is then updated using the Eq. (15).

15 $$\begin{aligned} P_i(X) = P_{i-1}(x) + \rho . Tree_i(X) \end{aligned}$$

where $\rho$ is the learning rate used.

#### Light gradient boosting machine

Light Gradient Boosting Machine (LightGBM) is also a kind of gradient boosting framework that was developed by Microsoft. This is faster and more memory efficient when compared to other Gradient Boosting methods like XGB. The reason behind this is that LightGBM uses a special type of splitting called as histogram splitting. Also, the tree is constructed leaf-wise instead of level-wise, as shown in the Fig. 7b. The information gain of each leaf node is calculated, and the one with the maximum information gain is further split. This improves the efficiency of training since deeper and more optimized trees are generated within a few splits. The residual calculated in this method is called a pseudo-residual and is given by the Eq. (16)

16 $$\begin{aligned} r_i = \frac{\delta L(y, P_{i-1}(x))}{\delta P_{i-1}(x)} \end{aligned}$$

where $L(y, P_{i-1}(x))$ is the Loss function, which may be RMSE or LogLoss, and $P_{i-1}(x)$ is the prediction output of the previous iteration. RMSE is used in case of regression problems and LogLoss is used in classification problems. Using these residuals calculated, the Boosting step is carried out. Then histogram-based binning is performed for the features. Overfitting issues are handled by the L1 and L2 regularizations. Though there are advantages, this method is prone to overfitting due to the leaf-based deep tree construction. This is also not very suitable for small data sets.

#### Extreme gradient boosting regression

**Fig. 8:**
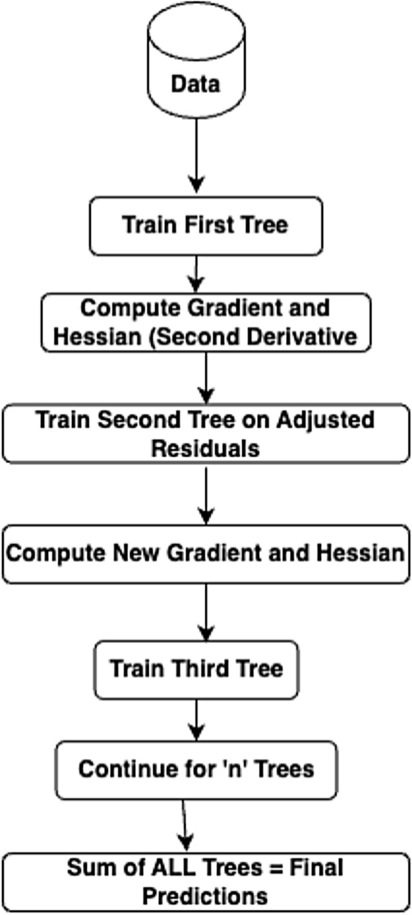
Graphical structure of extreme gradient boosting regression.

Extreme Gradient Boosting Regressor (XGBRegressor) is a modified version of Gradient Boosting Regressor, which can be implemented using various regulation techniques, including L1 and L2 regulation,the dropout method and early stopping. This method can reduce either over-fitting or under-fitting by reducing the value of the regulation coefficient^47^. The major advantage of this model is that it can perform pre-processing of each node in a parallel manner, hence saving time and landing with a faster prediction rate. XGB Machines can also automatically understand the imputations. When the complexity of the model goes high, regularization techniques like LASSO (L1) and Ridge (L2) are required to penalize it. The graphical structure is as shown in Fig. 8. The parameters required for regularization are as follows.

- *gamma* ($\gamma$): This specifies the minimum reduction in loss allowed for the number of splits. When the value of gamma is high, then the number of splits is fewer and vice versa.
- *alpha* ($\alpha$): This parameter is related to the L1 regularization and is on leaf weights. When this value is higher, there will be more regularization, which means that many leaf weights of the base learner are made zero.
- *lambda* ($\lambda$): This parameter is related to the L2 regularization and provides a smooth decrease in leaf weights unlike L1 which has hard constraints.

Multiple parameters help for building the trees in the regression, namely similarity score, gain, pruning criteria, etc. These parameters can be calculated as follows. In the Eq. (17), $\rho$ represents the similarity score, $r_i$ represents the residuals, and *n* represents the number of residuals. This parameter helps in growing the trees.

17 $$\begin{aligned} \rho = \frac{\sum _{i=1}^{n}r^2_i}{n} + \lambda \end{aligned}$$

For deciding on how to split the data, the parameter gain can be utilized and can be calculated by using the Eq. (18).

18 $$\begin{aligned} \eta = \rho _l + \rho _r - \rho _{root} \end{aligned}$$

where $\eta$ is the gain, $\rho _l$ is the similarity score of left tree, $\rho _r$ is the similarity score of right tree and $\rho _{root}$ is the similarity score of root. The tree is then pruned based on the value obtained using Eq. (14). If the value calculated is positive, then the tree is not pruned; if the result is negative, then the tree continues to be pruned further.

#### Linear regression

Linear Regression is a model that does the prediction by having an assumption that all variables are linked to each other under a straight-line relationship. Usually, the variables are considered to be independent and dependent. The relationship between these variables is calculated using a linear function. When the number of independent variables is one, then it is called as linear regression. The best fit straight line can be calculated using the Eq. (19), which is a traditional slope-intercept equation.

19 $$\begin{aligned} m_j = \beta _0 + \beta _1n_j \end{aligned}$$

where $m_j$ is the dependent variable, $n_j$ is the independent variable, $\beta _0$ is the constant and $\beta _1$ is the slope intercept. The performance of a Linear Regression model can be assessed using metrics like Coefficient of Determination ($R^2$), Root Mean Squared Error (RMSE) and Residual Standard Error (RSE). The Coefficient of Determination can be represented by Eq. (20), where $R_s$ is the residual sum of squares and $T_s$ is the total sum of squares.

20 $$\begin{aligned} R^2 = 1 - \frac{R_s}{T_s} \end{aligned}$$

The residual sum of squares can be calculated using Eq. (21), and the total sum of squares can be given by Eq. (22).

21 $$\begin{aligned} R_s= & \sum _{i=1}^{z}(m_j - \beta _0 - \beta _1n_j)^2 \end{aligned}$$

22 $$\begin{aligned} T_s= & \sum (m_j - m^*)^2 \end{aligned}$$

where $m^*$ is the mean of all the data points *j*.

### Stacking

**Fig. 9:**
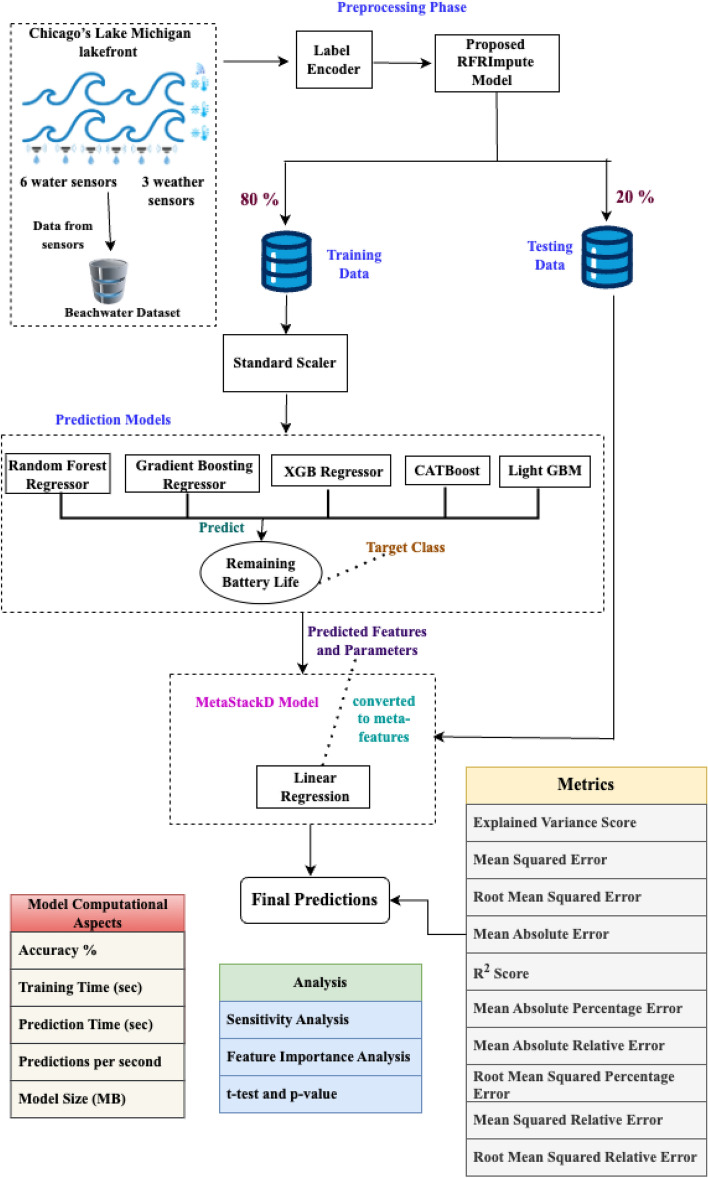
Proposed MetaStackD deep ensemble model for sensor battery life prediction.

Stacking is a powerful fusion method that combines the predictions of various base models to improve the performance of the final prediction. This is also called Stacked Generalization and is illustrated in Fig. 9. Stacking primarily focuses on providing the individual predictions of several base models or learners as input to the higher-level model termed “meta-model” or blender^48^. This meta-model finally combines or blends to provide a final prediction that is more accurate. Though traditional ensemble methods like bagging and boosting can be utilized, meta-learning provides a blended framework that leverages the advantages and performance of multiple models to produce an improved model. The meta-model learns to optimize the parameters and helps in generalization. This can also provide better model diversity. Meta-learning is best suited when the data sets are heterogeneous. Since we are focusing on developing a generalized framework for utilizing in the IoE environment, this is the best strategy to capture different complex aspects and relationships of the data. The steps involved in the procedure are data preparation, selection of model, training using the base models, prediction result on the validation data set, development of meta-model, training the meta-model, final predictions and evaluation of the model. Initially, the data set is prepared by cleaning and identifying the relevant features. Then, the entire data is subdivided into training and validation sets. Then, different models are selected to be used as base models for the ensemble approach. While selecting the models, various factors like diversity, hyper-parameters, type of errors, etc., are taken into account. Then the chosen base models are trained on the training set for learning the features. Once learning is complete, the validation data set is used for making predictions. Then, a meta-model is developed which is sometimes called a meta-learner that takes the predictions of the lower-level model as input and makes the final prediction. Any model like Linear Regression, Logistic Regression or Neural Network can be used for this purpose. The finalized meta-model is used for training the predictions obtained from the validation set. The initial predictions made by the base models are considered to be the features of the meta-model. The meta-model is then used for predicting the test set. Stacking generally produces an improved accuracy when compared to utilizing a single model.

Applying the above methodology, we performed experiments to test the predictive ability of our MetaStackD model. The following section describes the results, comparing model accuracy, efficiency, and relative performance with baseline models.

## Proposed methodology

The total power required for any IoT device or system completely depends on the type of application in which the device is deployed. Usually, the sensors deployed in the IoT device, require limited energy during sleep mode since no data or limited data is shared when compared to active mode where huge data is shared rigorously. But, in real-time some sensors require equal energy all the time. The power consumption depends on all the individual components of the IoT system. The sensors in the IoT device get power and function with the help of rechargeable, non-rechargeable or disposable batteries. The type of battery to be used for deployment can be chosen based on three factors, which are listed below.

- The total amount of electrical discharge, waste and performance.
- The amount of energy used due to self-discharge, which means that the energy utilized even during idle state.
- Physical features of the battery and safety.

The batteries are further selected based on the type of application for which they are going to be used. When the application is going to consume less power, disposable batteries can be used in the device. When the power consumption is high, rechargeable batteries can be utilized. Based on the type of battery used, the power consumption, as well as the lifetime, can be estimated using techniques like software-based estimation and short-term power measurements. Software-based estimations cannot be practically used in real-time, as the test device has to be attached while measuring. Hence, to continuously estimate the battery life, this method cannot be used. AI techniques can be used for predicting the battery’s lifetime in the real world as there is no requirement for any devices or resistors as in physical estimation techniques. This section discusses the proposed methodology for periodically predicting the lifetime of the sensor battery in an IoT environment without any manual intervention.

**Algorithm 1:**
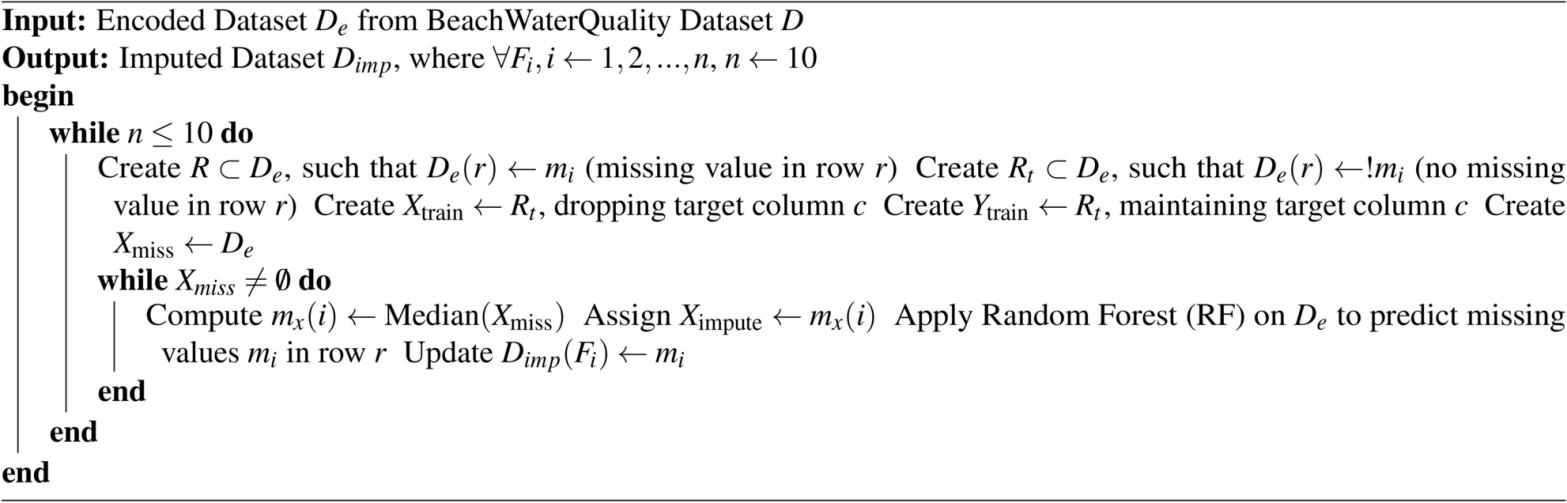
Proposed RFRImpute for handling missing values

The proposed framework is illustrated in Fig. 9 and can be used for any IoE environment to predict the lifetime of the sensors’ battery after deployment in real-time. All IoE applications are based on the sensor data, and these sensors are always battery-operated. Since the proposed framework is for predicting the life of a battery for any sensor network, it can be used in any smart IoE application though tested in the marine environment. In real-time, the smart devices are deployed, and the sensors start to perceive the environment. When they start transferring the data sensed, the sensor starts to consume power from the battery. Hence, the remaining lifetime of the sensors’ battery needs to be estimated so that the battery doesn’t drain out, thereby missing data from the sensor. When the sensor battery gets drained unexpectedly, there is a chance of missing important data, which can lead to drastic issues. This issue can be overcome by using the proposed framework. The framework consists of various components starting from initial preprocessing, encoding, training and prediction. Finally, the framework is validated. The data acquired from the IoT device is first analyzed to check if any missing values are seen. The missing values are filled using the proposed RFRImpute algorithm. This algorithm uses an ML model to predict the missing values. The predicted missing values are then updated in the data set. The algorithm is applied for every feature with missing values. Once all missing values are filled in the data set, the iteration is stopped. Before applying the RFRImpute algorithm, which is shown in Algorithm 1, the data set is tested for categorical values and the entire data set is encoded using the Label Encoder method. The pre-processed data is now split into training and testing data sets using the 80–20 rule. The reason for choosing 80% training data is to ensure that the model learns all the patterns perfectly from the data. Also the 80–20 rule helps to maintain a good balance between bias and variance. Also it maintains a statistical consistency among the splits.

**Algorithm 2:**
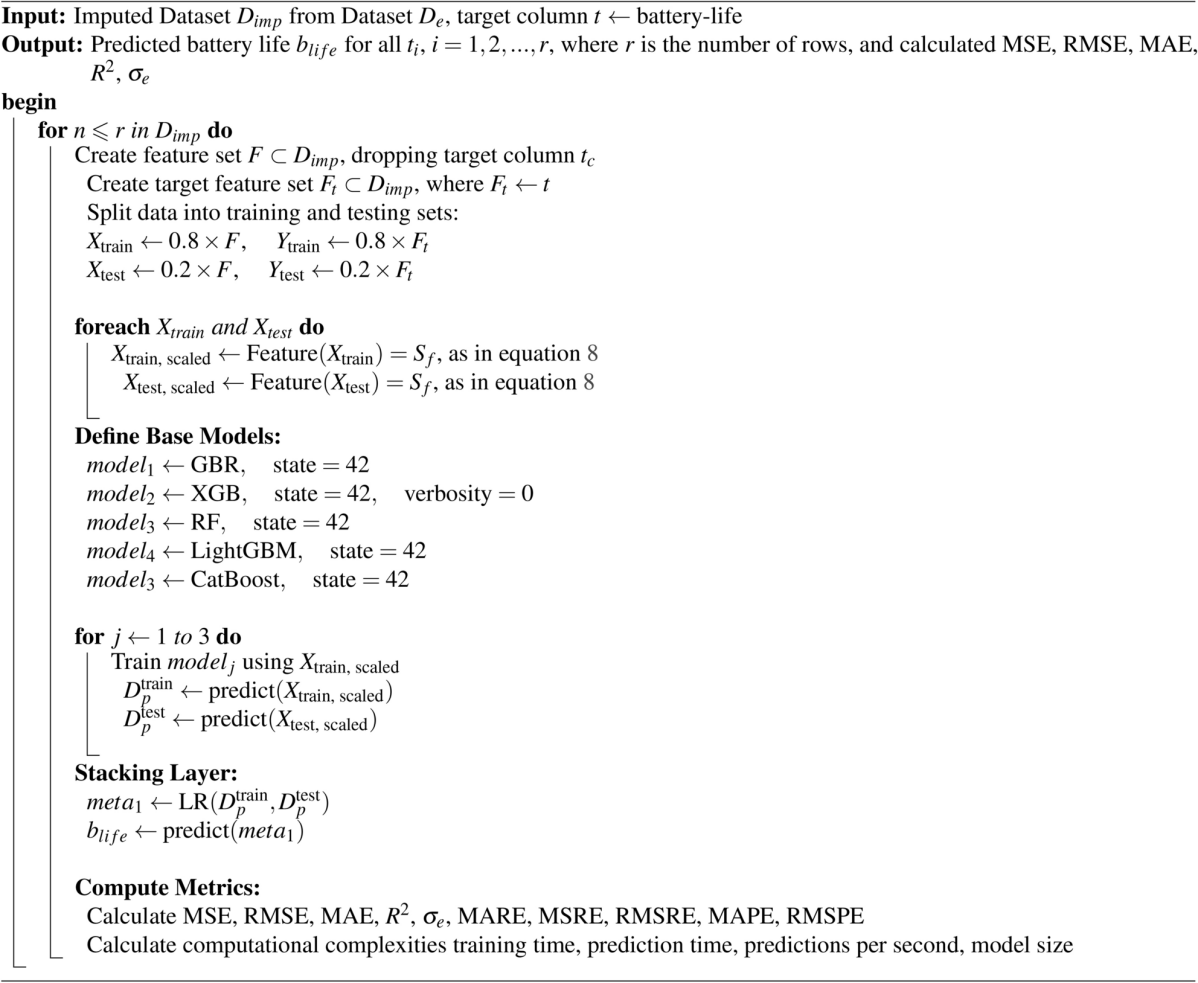
Proposed MetaStackD ensemble model

The proposed MetaStackD algorithm shown in Algorithm 2 is a robust meta-learner that predicts the lifetime of the smart device’s sensor battery in a short period. This helps the maintenance engineers in scheduling the replacement of the sensors’ batteries right time so that no real-time data perceived from the environment is lost. The preprocessed data is then standardized using the Standard Scalar methodology before the training phase. The five base learners utilized for the initial level of training and prediction of the battery life are RF, GBR, XGB, LightGBM and CatBoost. Before the preprocessed training data is utilized for learning using these base models, the features are standardized. The standardization process is exempted in the case of the CatBoost model alone. When all the base learners have learnt, the validation data set is utilized for the prediction of the target class namely battery life. A5-fold cross-validation is performed. These prediction parameters and features are used for the construction of the meta-features. Using the weak learner, which is the Linear Regression model, the final predictions are made. The proposed framework is validated using evaluation metrics like Mean Absolute Error (MAE), Mean Squared Error (MSE), Root Mean Squared Error (RMSE), Coefficient of Determination ($R^2$), Mean Absolute Relative Error (MARE), Mean Squared Relative Error (MSRE), Root Mean Squared Relative Error (RMSRE), Mean Absolute Percentage Error (MAPE), Root Mean Squared Percentage Error (RMSPE) and Explained Variance Score ($\sigma _e$). For further justification, computational aspects are also taken into account. The computational aspects like training time in seconds, prediction time in seconds, prediction speed in terms of several observations per second and size of the model are computed. Also, to claim the robustness of the model, feature importance analysis and sensitivity analysis are performed. Further, the framework is designed in such a way that the model learns all the non-linear relationships in real time and can adapt itself to any application by the usage of meta-learning aspects.

The experimental setup, the data set used for training as well as validation, metrics, their interpretations and analysis are discussed further in detail in the next section.

Our experimental results show the efficiency and reliability of the MetaStackD model for predicting battery life in IoE settings. The last section provides an overview of the important contributions of this work and considers possible future directions for further enhancement.

**Table 2:** Deployment details of sensors in Chicago Park District.

Name of the sensor	Type of sensor	Longitude	Latitude
63 Street Beach	Water	− 87.571453	41.784561
Calumet Beach	Water	− 87.527356	41.714739
Montrose Beach	Water	$-$ 87.638003	41.969094
Ohio Street Beach	Water	− 87.613083	41.894328
Osterman Beach	Water	$-$ 87.651008	41.987675
Rainbow Beach	Water	− 87.550081	41.760147
63 Street Weather Station	Weather	− 87.572619	41.780992
Foster Weather Station	Weather	− 87.647525	41.976464
Oak Street Weather Station	Weather	− 87.622817	41.901997

## Results and discussion

The proposed framework is experimented with using the real-time IoT system deployed in the Chicago district. This section discusses in detail the data set used for validating the proposed methodology, the results obtained when the proposed RFRImpute pre-processing algorithm is used, the prediction made by ensemble base learners and the final meta-model’s prediction. This also discusses the robustness of the proposed MetaStackD model and how it can be helpful in the real-time deployment of IoE applications.

### Data set description

The proposed algorithms RFRImpute and MetaStackD are validated using a real-time bench-marked data set available at https://catalog.data.gov/dataset/beach-water-quality-automated-sensors. Chicago Park District provides this data set^49^. The data is retrieved using sensors located at the beaches along Chicago’s Lake Michigan lakefront using both water and weather sensors. The sensors are deployed to sense the beach data every hour during summer and other seasons. The details about the various sensors deployed and their locations are tabulated in Table 2. The sensors are deployed at nine locations out of which six are water sensors and three are weather sensors. The data set has ten features, namely beach name, the timestamp of measurement, the temperature of water, turbidity, transducer depth, height of the wave, period of the wave, battery life, measurement ID and label. The type of value sensed as the feature is given in detail in Table 3. There are around 39.5k data sensed by the IoT environment.

**Table 3:** Sensor data perceived.

Feature	Description	Unit	Data type	Data range
Beach Name	Name of the beach where the sensor is deployed	String	String	9 locations
Measurement Timestamp	Sensor senses the environment in a hourly basis during summer	Date	Date	30/8/2013 8 am to 14/1/2025 11 am
Water Temperature	Temperature of water	Celcius	Float	9.1 to 31.5
Turbidity	Waves’ turbidity	NTU	Float	0.01 to 1683.48
Transducer Depth	Depth	Meters	Float	− 0.082 to 2.214
Wave Height	Water waves’ height	Meters	Float	0.013 to 1.467
Wave Period	Period of waves	Seconds	Integer	1 to 10
Battery Life	Remaining life of the sensors’ battery	Voltage	Float	4.8 to 13.3
Timestamp Label	Integration of time and date in 24 hours schedule	Date and time	String	2013-08-30T08:00:00 to 2025-01-14T11:00:00
Measurement ID	Beach name followed by the unique ID for measurement	String	String	Not Applicable

### Pre-processing

In this step, the data set was inspected to verify if any noise, abnormalities and missing values were present using visualization plots like scatter plots, bar plots, heat maps etc. During basic statistical visualization, it was found that there were a total of 10 features, out of which the “battery life” feature was the target feature. Out of the 10 features, there were missing values for 8 features, and the details are as shown in Table 4. Before filling in the missing values, since the data set had categorical features, the Label Encoder mechanism was used for encoding the data. Then the encoded data is used as input for the algorithm RFRImpute to fill in the missing values. The algorithm is applied iteratively on each feature to fill all the missing values and finally, the filled-up data set is used for further process. No outliers or noise were seen in the data set.

**Table 4:** Missing values in data set.

Features	Total No. of missing values
Beach Name	0
Measurement Timestamp	6
Water Temperature	6
Turbidity	6
Transducer Depth	24889
Wave Height	233
Wave Period	233
Battery Life	6
Timestamp Label	6
Measurement ID	0

**Fig. 10:**
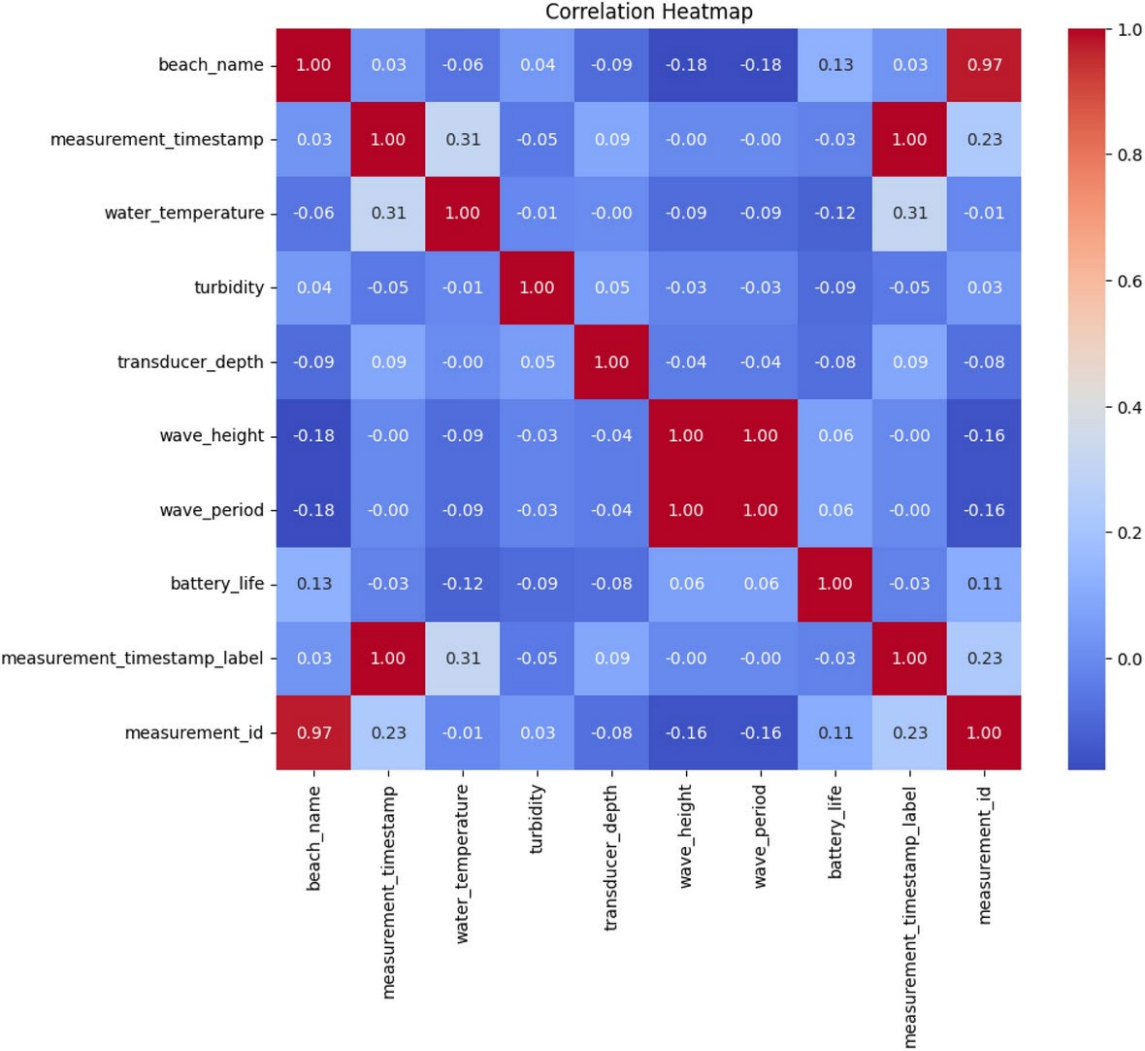
Correlation heat map: visualization of the relationship between the features.

The correlation heatmap obtained is shown in Fig. 10, which is generated after the proposed imputation algorithm is applied to the data set. From Fig. 10, it is evident that almost all features are highly correlated and have a negative correlation. The pre-processed data are then subdivided into training and testing sets using the 80–20 rule. The features are then standardized using the standard scalar method.

### Prediction models

The scaled and standardized features are then utilized for learning. The models were experimented with using the Google Colab NVIDIA T4 version with 16GB GDDR6 and 320 Tensor Cores version. The training data are trained using the prediction models, namely the random forest regression model, gradient boosting regression model, extreme gradient boosting regression model, light gradient boosting machine and categorical boosting,g individually and the prediction results are obtained. Then, these models are utilized as base learners for ensemble learning. Initially all the basic voting mechanisms like simple averaging, weighted average and median ensemble are used to predict the final battery life. Then, the prediction results are obtained using the proposed MetaStackD Model. The results obtained with individual base learners and the one with a voting mechanism are then compared with the proposed MetaStackD Deep Ensemble Model. This section discusses in detail the results obtained at each stage.

#### Random forest regressor model

The scaled training data set is trained using the random forest regressor model with 100 estimators and 42 as a random state. The parameter 100 estimators represent that 100 decision trees are constructed in the ensemble. Randomness 42 is a standard setup done for maintaining randomness while constructing the trees. The RF model learns the features, and the learner is used for predicting the target variable “battery life”. The performance metrics are calculated along with confidence intervals (CI) and standard deviations (SD). This is done to assess the variability. The validation of the model with the testing set gave a prediction result with the performance of MSE 0.009486, RMSE 0.09739, MAE 0.02574, $R^2$ 0.9837 and explained variance 0.9837. All the metrics are tabulated in Table 5, showing the CI and SD. The interpretations of the metrics over the model are also highlighted in the Table 5. The key takeaway from the analysis was that the model performs well with a low error rate, high predictive power, and stable performance, but when the target values are relatively small, the model might struggle. The total training time required for learning the features using the RF model was 20.299 s, the prediction time required for predicting the target variable was 0.1305 s, the model size was 53.07 MB and the prediction speed was $5.351 * 10^4$ observations per second.

**Table 5:** performance metrics of random forest regression model.

Metric	Value	SD	95% CI	Interpretation
MAE	0.0257	0.0012	0.0235–0.0280	The predictions are deviating from the actual values by $\sim$0.0257
MSE	0.0095	0.0015	0.0068–0.0128	The squared errors are prone to huge mistakes than smaller errors
RMSE	0.0971	0.0078	0.0827–0.1133	This shows a low error
R⌃2	0.9837	0.0026	0.9783–0.9881	98.37% of variance is shown
MARE	0.0025	0.0001	0.0022–0.0027	Very low level of relative error
MSRE	0.0001	0.0000	0.0001–0.0001	extremely low squared relative error
RMSRE	0.0099	0.0008	0.0084–0.0115	Low error
MAPE	0.2465	0.0120	0.2238–0.2699	Around 24.65% of predictions are wrong
RMSPE	0.9918	0.0752	0.8444–1.1520	Higher variation in percentage errors

#### Gradient boosting regressor model

When the training data set was learnt using the Gradient Boosting Regressor model with 100 estimators, 42 random states and a learning rate of 0.1, the results obtained are shown in Table 6. The table also shows the CI and SD for each metric. The interpretations of the obtained value are also highlighted in the table. The very high MAPE suggests that the model is struggling significantly, and the high RMSPE shows that the model is unstable. The time required for learning the features of the training data was 5.8856 s, the time required for predicting was 0.008434 s, the prediction speed was $8.2823 * 10^5$, and the model size was 13.3 MB.

**Table 6:** Performance metrics of gradient boosting model.

Metric	Value	SD	95% CI	Interpretation
MAE	0.3326	0.0039	0.3250–0.3402	The predictions are deviating from the actual values by $\sim$0.3326
MSE	0.2100	0.0052	0.2002–0.2202	The squared errors are prone to huge mistakes than smaller errors
RMSE	0.4582	0.0057	0.4474–0.4693	This shows a moderate error
R⌃2	0.6388	0.0140	0.6102–0.6671	63.88% of variance is shown
MARE	0.0305	0.0004	0.0298–0.0312	Moderate level of relative error
MSRE	0.0018	0.0001	0.0017–0.0019	Low squared relative error
RMSRE	0.0428	0.0007	0.0415–0.0442	Moderate RMSRE value
MAPE	3.0501	0.0373	2.9794–3.1227	Around 305% of predictions are wrong
RMSPE	4.2840	0.0667	4.1550–4.4157	Model performs poorly with different magnitudes

#### Extreme gradient boosting regressor model

The results obtained when the training data is used for learning the features using the XGBRegressor model, initialized with the same number of estimators and random states as 100 and 42, respectively are tabulated in Table 7 along with CI and SD for each evaluated metric. The results are obtained with a learning rate of 0.1. The table also throws an insight into the interpretation of the model performance. The training time required for learning the features using this model was 0.123943 s, and the prediction time was 0.004286 s. The number of observations predicted per second when this model was utilized was 1.6297 * $10^6$, and the size of the model was 39.2 MB.

**Table 7:** Performance metrics of extreme gradient boosting model.

Metric	Value	SD	95% CI	Interpretation
MAE	0.1617	0.0025	0.1570–0.1667	The predictions are deviating from the actual values by $\sim$0.1617.
MSE	0.0695	0.0035	0.0630–0.0762	The squared errors are prone to huge mistakes than smaller errors
RMSE	0.2635	0.0067	0.2511–0.2761	This shows a lower error
R⌃2	0.8806	0.0070	0.8671–0.8931	88.06% of variance is shown
MARE	0.0148	0.0002	0.0143–0.0152	Moderate level of relative error
MSRE	0.0006	0.0000	0.0005–0.0006	Low squared relative error
RMSRE	0.0240	0.0006	0.0229–0.0251	Low RMSRE value
MAPE	1.4766	0.0230	1.4320–1.5209	Around 147.66% of predictions are wrong
RMSPE	2.3981	0.0580	2.2860–2.5092	Moderate error

#### Light gradient boosting machine

The training data set was trained for learning the features with the LightGBM initialized to construct 100 trees, a learning rate of 0.1 and a randomness of 42. The results obtained are shown in the Table 8. The total time required for learning the features was 0.177582 s, and the time for predicting the target battery life was 0.0141 s. The size of the model was 28.6 MB and the prediction speed was 4.953 * $10^5$ observations per second.

**Table 8:** Performance metrics of light gradient boosting machine.

Metric	Value	SD	95% CI	Interpretation
MAE	0.1354	0.0024	0.1309–0.1400	Has the lowest error compared to XGBoost and Gradient Boosting
MSE	0.0561	0.0039	0.0489–0.0644	Indicates fewer large errors
RMSE	0.2368	0.0083	0.2212–0.2537	Better predictive stability
R⌃2	0.9034	0.0073	0.8889–0.9169	90.34% of variance is shown by the model
MARE	0.0124	0.0002	0.0120–0.0129	Lower level of relative error
MSRE	0.0005	0.0000	0.0004–0.0006	Low squared relative error
RMSRE	0.0223	0.0009	0.0207–0.0242	Lowest RMSRE value
MAPE	1.2435	0.0225	1.2004–1.2897	Around 124.35% of predictions are wrong
RMSPE	2.2292	0.0873	2.0671–2.4169	Moderate error

#### Categorical boosting

The initial hyper-parameters were 0.1 learning rate, randomness to be 42 and 100 estimators. The results obtained when the model was trained and predicted are tabulated in Table 9. The time required for training was 0.239728 s, and for predicting the battery life was 0.001345 s. The total size required for deploying the model would be 11.6 MB, and it would predict at a speed of 5.193 * $10^6$ observations per second.

**Table 9:** Performance metrics of categorical boosting.

Metric	Value	SD	95% CI	Interpretation
MAE	0.2714	0.0035	0.2649–0.2785	Worse when compared to LightGBM and XGBoost
MSE	0.1589	0.0047	0.1501–0.1687	Worser than LightGBM and XGBoost
RMSE	0.3986	0.0058	0.3874–0.4107	Higher when compared to LightGBM and XGBoost
R⌃2	0.7267	0.0117	0.7034–0.7502	72.67% of variance is shown by the model
MARE	0.0249	0.0003	0.0243–0.0255	Worser than LightGBM and XGBoost
MSRE	0.0014	0.0001	0.0013–0.0015	Worse when compared to LightGBM and XGBoost
RMSRE	0.0374	0.0007	0.0361–0.0389	Worser than LightGBM and XGBoost
MAPE	2.4903	0.0327	2.4279–2.5545	Worser than LightGBM and XGBoost
RMSPE	3.74	0.0722	3.6109–3.8892	Worser than LightGBM and XGBoost

#### Proposed meta-model MetaStackD

The prediction results of base learners Random Forest Regressor, Gradient Boosting Regressor, Extreme Gradient Boosting Regressor, Light Gradient Boosting Regressor and Categorical Boosting were utilized for creating the meta-features. The predictions of training and testing data are appended to a new set of features. These are used as train-meta-features and test-meta-features for the meta-model. The linear regression model, which is a weak learned, r is used as the meta-model for learning the meta-features, which are built in the previous step. The reason for choosing Linear regression is that it provides clear as well as interpretable coefficients for understanding how much each base model contributes to the final prediction. Other complex models like neural networks can overfit the meta-features easily, which leads to very poor generalization. Moreover, it is faster and computationally inexpensive. The train-meta-features are learnt using the meta-model initially. Then after the learning phase, the test-meta-features are used for getting the final predictions. The proposed MetaStackD model gave the final predictions whose evaluation metrics showed an improved result as tabulated in the Table 10.

**Table 10:** Performance metrics of stacking meta model.

Metric	Value	SD	95% CI	Interpretation
MAE	0.0286	0.0011	0.0266–0.0307	Identical to Random Forest
MSE	0.0094	0.0014	0.0070–0.0124	Outperforms Random Forest in a marginal line
RMSE	0.0967	0.0074	0.0837–0.1114	Outperforms Random Forest in a marginal line
R⌃2	0.9838	0.0025	0.9784–0.0025	98.38% of variance is shown by the model
MARE	0.0027	0.0001	0.0025–0.0030	Identical relative error as Random Forest
MSRE	0.0001	0.0000	0.0001–0.0001	Better than Random Forest
RMSRE	0.0098	0.0007	0.0085–0.0112	Better than Random Forest
MAPE	0.2733	0.0110	0.2521–0.2954	Slightly worser than Random Forest
RMSPE	0.9845	0.0699	0.8537–1.1234	Better compared to Random Forest

**Fig. 11:**
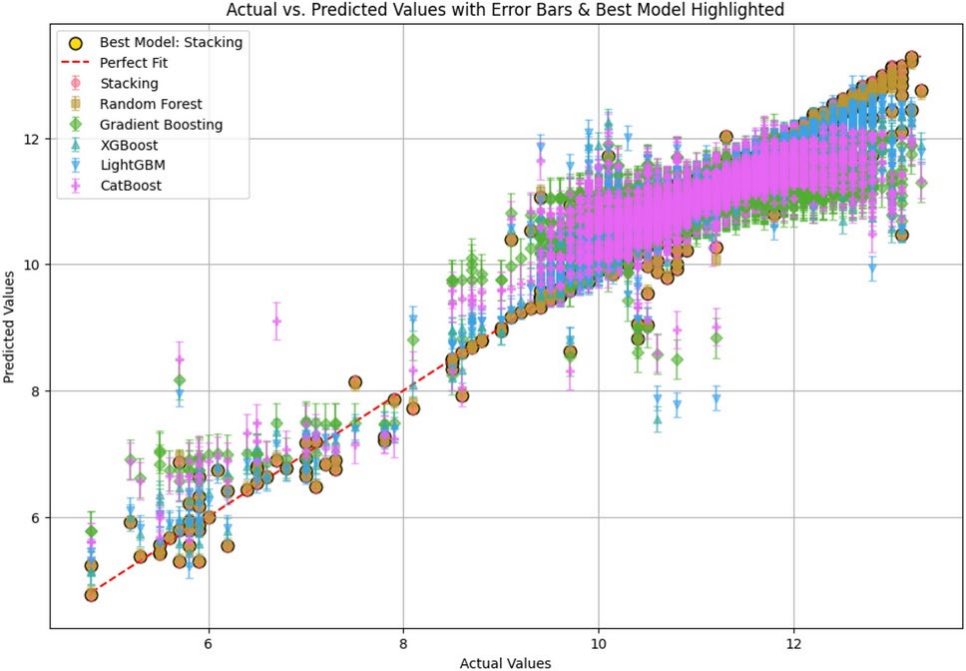
Comparison of actual vs predicted values: all base learners and proposed MetaStackD.

The time required for training using the proposed model was 0.003796 s. The time required for predicting the final battery life of the sensors was 0.001019 s. The predicted battery life compared with the actual battery life of the sensors for all the base learners and the proposed MetaStackD model is illustrated in Fig. 11. From Fig. 11 it is very clear that the proposed MetaStackD model is the best model when compared to the standalone base models. The size of the proposed MetaStackD model is 5.3 MB.

**Fig. 12:**
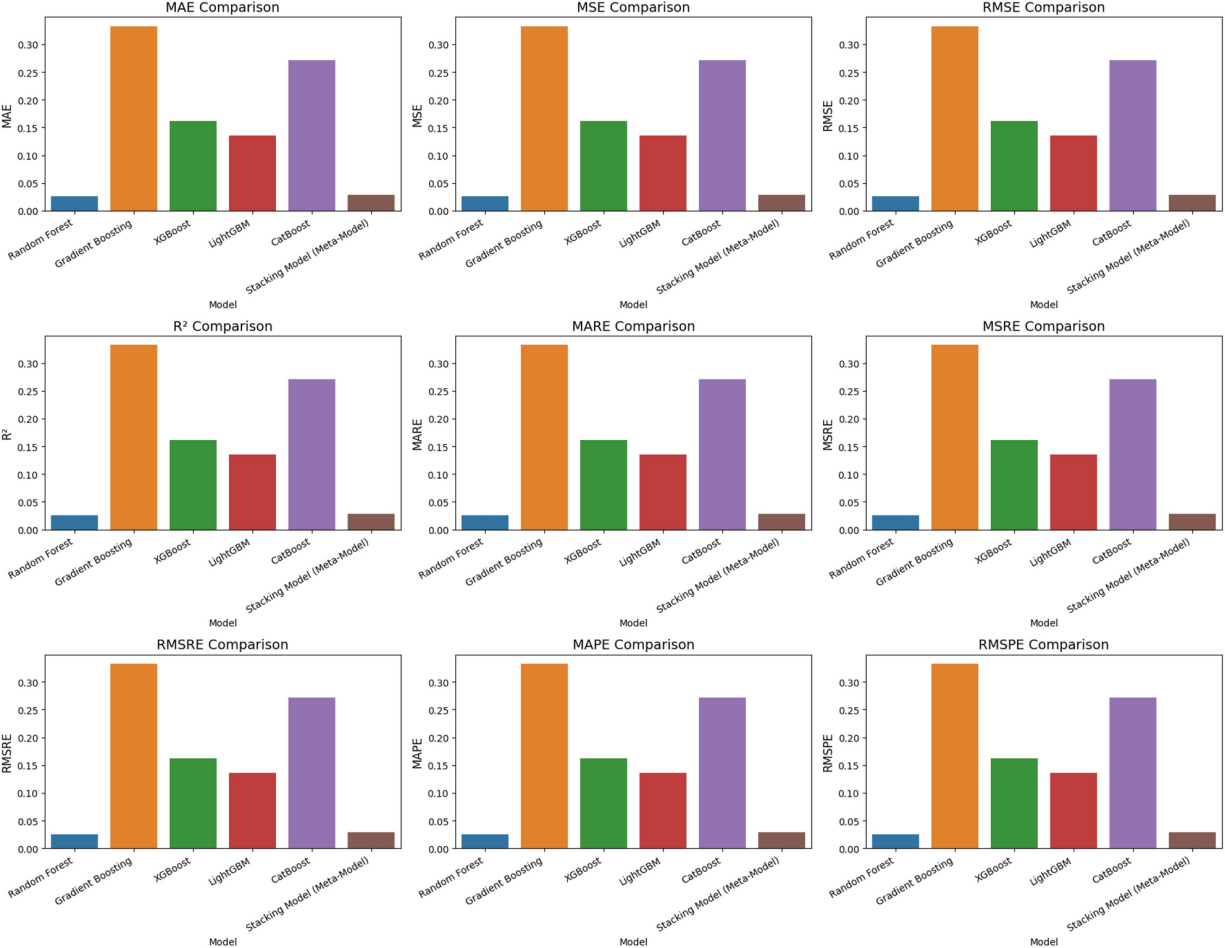
Comparison of performance metrics: all standlone base learners vs proposed MetaStackD.

The Fig. 12 shows the comparison of the performance metrics of all standalone base learners with the proposed MetaStackD model. The comparative plot clearly shows that the proposed MetaStackD performs better in the case of error metrics MSE and RMSE when compared to the standalone base learner Random Forest, which is the best individual model as per the experiments conducted. MetaStackD has the highest $R^2$ score which specifies that the model explains a high level of variance concerning the target variable. Concerning the relative error metrics MSRE and RMSRE, MetaStackD performs better when compared to the standlone best model RF. In the case of the percentage-based error metrics RMSPE, MetaStackD performs slightly better than standalone RF.

#### Voting ensemble learning model

The base learners discussed in the previous subsections were utilized for constructing a voting-based ensemble model. The predictions of the base models are ensembled using all three types of voting regressors, namely Simple Averaging, Weighted Averaging and Median ensemble. Three stack models were generated using the voting ensemble learners. The target feature, namely battery life, was predicted using the final voting models, and the results obtained are tabulated in Table 11.

**Table 11:** Performance metrics of voting ensemble models vs MetaStackD.

Model	MAE	MSE	RMSE	R⌃2
Voting (Averaging)	0.1194	0.0371	0.1926	0.9362
Voting (Weighted)	0.1194	0.0371	0.1926	0.9362
Voting (Median)	0.0895	0.03	0.1734	0.9483
MetaStackD	0.0285	0.0093	0.0965	0.9839

**Fig. 13:**
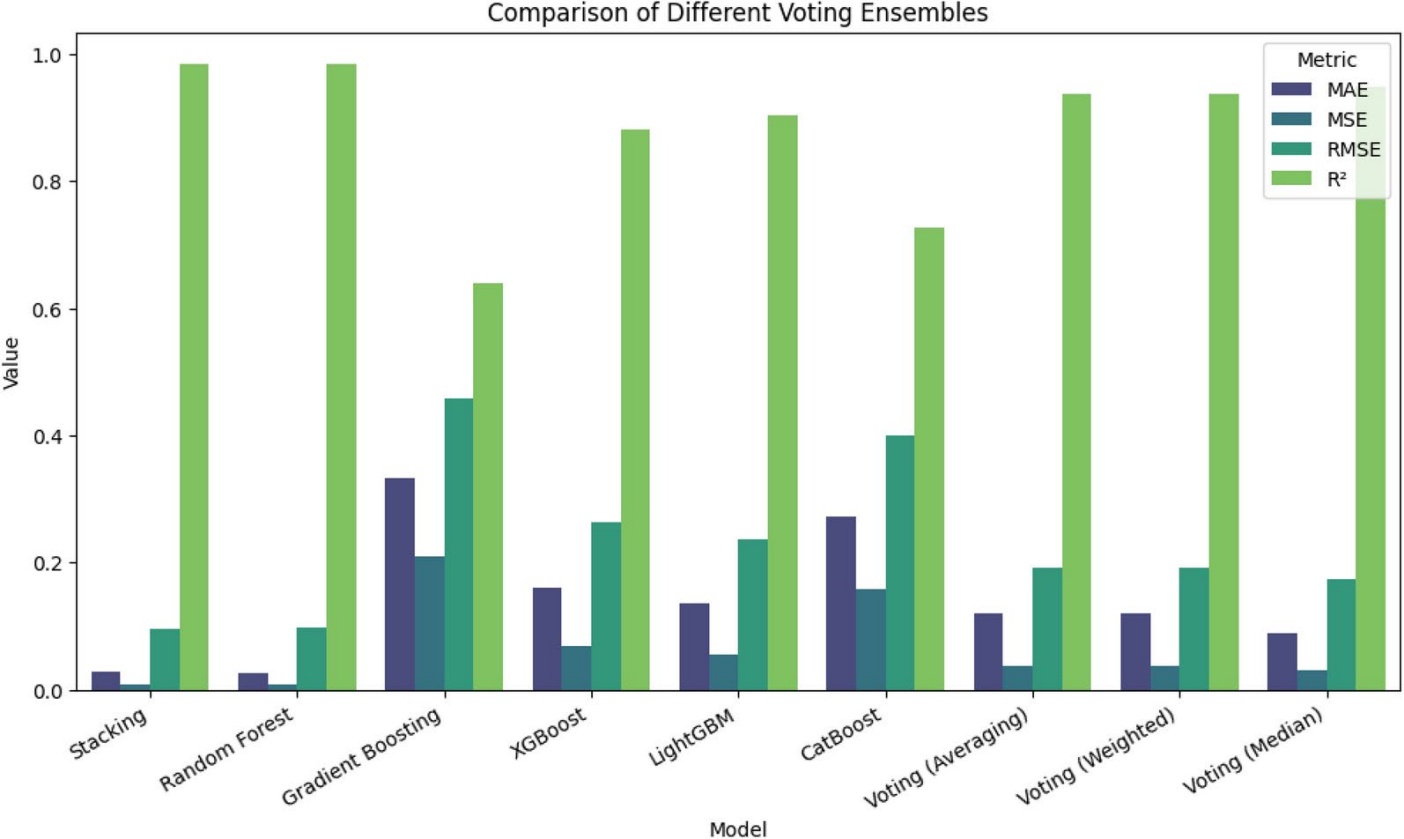
Comparison of performance metrics: standalone models vs MetaStackD and voting ensembles.

From the Table 11, it is very clear that the MetaStackD achieves the lowest MAE, MSE, RMSE and highest $R^2$. This suggests that MetaStackD effectively enhances the prediction accuracy compared to the voting models. The MetaStackD has the lowest average prediction error which states that it provides a very precise estimate. The non-linear and complex relationships are perfectly captured by the proposed MetaStackD model, which the simple voting methods might miss. The performance metrics and results obtained by standalone models, proposed MetaStackD and the three variations of Voting ensemble models are plotted in Fig. 13. The plot 13 compares the four key regression metrics, namely MAE, MSE, RMSE and $R^2$. The MAE is represented by a dark blue colour in the plot 13 and when the value is lower, it is considered to be better. Hence MetaStackD model is better when compared to standalone models and voting-based ensembles. Also, concerning MSE and RMSE, the lower value is better and is represented by blue and teal in the plot 13. MetaStackD achieves the highest $R^2$ and is represented by green in the plot 13, and higher is better. From all these analyses, it is evident that the proposed MetaStackD is the best choice due to the lowest error rates and highest $R^2$.

### Discussion on results

Our proposed algorithm RFRImpute for pre-processing helps in handling the missing values with improved performance as the regression model is utilized for predicting the missing values based on the available values in the data set instead of traditional statistical measures. The meta-learning-based ensemble model MetaStackD outperforms all the traditional standalone prediction models like RF regressor, GB Regressor, XGB Regressor, LightGBM and CatBoost as well as voting-based ensembles. The experimental analysis clearly shows that the time required for training, as well as prediction, is reduced when the proposed framework is used. Table 12 represents the results obtained as a result of the Wilcoxon signed-rank test, which is a non-parametric statistical test for comparing paired samples to determine if there is a significant difference. The test was conducted having the proposed MetaStackD model as a reference and compared against all other models under experimentation. From the p-value obtained, the statistical significance can be determined. As the p-value (0.000532) is less than 0.05 in the case of voting, the proposed MetaStackD outperforms the voting models in a significant way. The p-value for MetaStackD vs Gradient Boosting specifies that the proposed model shows improved performance. Similarly, XGBoost and CatBoost are weaker than MetaStackD since the p-values are 0.000277 and 0.037910, respectively.

**Table 12:** Wilcoxon signed-rank test results.

Reference model	Compared model	Wilcoxon statistic	p-Value
MetaStackD	Voting	11615447.0	0.000532
	Random Forest	12197496.0	0.991448
	Gradient Boosting	11624091.0	0.000643
	XGBoost	11586574.0	0.000277
	LightGBM	11941825.0	0.126591
	CatBoost	11849440.0	0.037910

Though from the tests, it was shown that RF and LightGBM performed similarly to MetaStackD, our proposed model shows better results in error metrics and computational aspects, which is evident from Table 13. The proposed MetaStackD has the least size of 5.3 MB, which is best for deploying in real-world smart IoE application,s and hence, scalability issues can also be handled efficiently. It is evident from the Table 13 that concerning the other computational metrics, MetaStackD performs better when compared to the other models. To gain a deep understanding of the importance of the features, we performed a sensitivity analysis.

**Table 13:** Comparison of computational aspects: all models vs proposed MetaStackD.

Model	Training time (s)	Prediction time (s)	Prediction speed Observations/sec	Model size (MB)
Random Forest	20.299	0.1305	5.351 * $10^4$	53.07
Gradient Boosting	5.8856	0.00843	8.2823 * $10^5$	13.3
XGBoosting	0.123943	0.00428	1.6297 * $10^6$	39.2
LightGBM	0.177582	0.0141	4.953 * $10^5$	28.6
CatBoost	0.239728	0.00134	5.193 * $10^6$	11.6
Voting (Averaging)	27.6053	0.4768	1.4648 * $10^6$	105.51
Voting (Weighted)	27.1993	0.7152	9.7657 * $10^4$	105.51
Voting (Median)	136.6324	0.2362	1.6327 * $10^3$	10.4
Proposed MetaStackD	0.003796	0.001019	6.8563 * $10^6$	5.3

The results obtained as a result of the sensitivity analysis are tabulated in Table 14. In Table 14, the column Mean Sensitivity provides the average importance across all the standalone base models and the proposed MetaStackD model. This metric indicates the consistency of the feature in contributing to the prediction of the target battery life. Among all the features in the data set, the most important features are turbidity, measurement ID and measurement timestamp. The moderately important features are wave height and transducer depth whereas the least important features are water temperature and wave period across all models. Moreover, almost all the models ignored the feature named beach name and this indicates that this feature is not useful in prediction.

**Table 14:** Summary of sensitivity analysis across models.

Feature	Random forest	Gradient boosting	XGBoost	LightGBM	CatBoost	MetaStackD	Mean sensitivity
turbidity	0.0437	0.2691	0.2297	0.2272	0.13877	0.1817	0.181713
measurement_id	0.2621	0.0889	0.1551	0.1726	0.03604	0.1429	0.142959
measurement_timestamp	0.1452	0.0472	0.2231	0.2229	0.07361	0.1424	0.142417
measurement_timestamp_label	0.1491	0.06486	0.00001	0.0000	0.07701	0.0582	0.058187
wave_height	0.0455	0.01698	0.0703	0.0789	0.04322	0.0510	0.051002
transducer_depth	0.0241	0.01241	0.0239	0.0421	0.02319	0.0252	0.025172
wave_temperature	0.0045	0.0086	0.0101	0.0153	0.01057	0.0098	0.009822
wave_period	0.0078	0.0022	0.0019	0.01516	0.00021	0.0055	0.005469
beach_name	0.000015	0.00001	0.00001	0.00001	0.000000	0.000003	0.000003

**Fig. 14:**
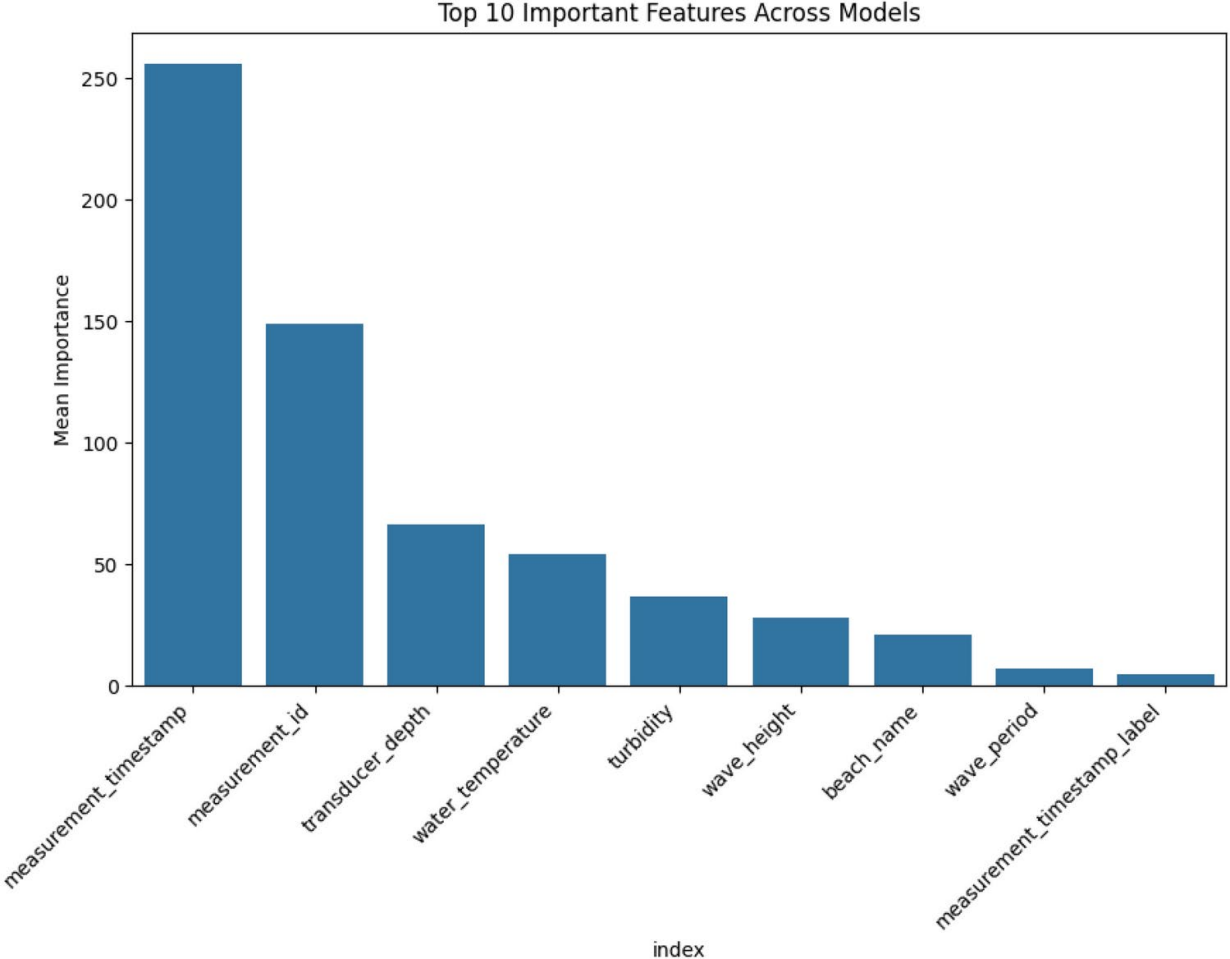
Ranking of the features based on Feature Importance Across ALL Models.

Similar to the sensitivity analysis, the feature importance scores were also estimated and compared across all models and the comparative analysis plot is shown in Fig. 14. The mean feature importance is estimated by averaging the importance score of each feature across all the standalone base models. This provides a rank on the features, which helps in understanding how influential the feature is. The highest mean feature importance score was 256.00, which corresponds to the feature measurement timestamp. The lowest mean importance score obtained was 4.4,0 which corresponds to the measurement timestamp label. Based on the scores, measurement timestamp and measurement ID are the most important features, as illustrated in Fig. 14. Wave period and measurement timestamp labels are considered to be the least important feature. The highest importance of the measurement timestamp indicates that the data is strongly time-dependent. The physical factors measurement id, transducer depth and water temperature also show high importance as they affect the water quality. The temporal details and cycles of waves seem to have a minimal impact on predictions. The findings from this feature importance analysis suggest that environmental conditions are more critical than the location and wave cycles.

The contributions of the proposed RFRImpute algorithm for pre-processing in the framework are as follows.

- The label encoding scheme utilized for encoding the categorical variables is computationally efficient. It is faster when compared to other encoding techniques like one-hot encoding. It is also space-efficient. The reason behind the efficiency in space is that, unlike the one-hot encoding scheme, this scheme uses a single column to represent all the categorical values. One-hot encoding uses binary columns, thereby increasing the dimensionality of the data set and hence requiring more space. Moreover, the label encoding scheme preserves the ordinal order of the categorical values in the data set. This is best suited for tree-based models as no linear relationship is considered.
- The RFRImpute algorithm uses RF as the imputed model for the prediction of the missing values. Since label encoding is used, the dimension is not increased, and when the missing values are dropped as in traditional approaches, the feature space might get reduced hence affecting the accuracy. To maintain the feature space and to improve the accuracy, instead of utilizing statistical measures, the RF model is used.
- From the correlation heat map, it was visible that there was intricate dependency among the data. Moreover, the relationship was non-linear. Hence, for handling such relationships, a robust methodology is required, and RF is the right choice for this scenario.
- The data set was characterized by a combination of numerical and categorical data. Model-based imputation is the best choice for handling such data.
- The algorithm also provides a feature importance score during the imputation process. This helps in handling multivariate relationships.
- The algorithm also reduces the over-fitting issues, thereby giving generalized imputation.

The contribution of the proposed MetaStackD Ensemble model for the prediction of sensors’ batteries in any IoE environment is as follows.

**Fig. 15:**
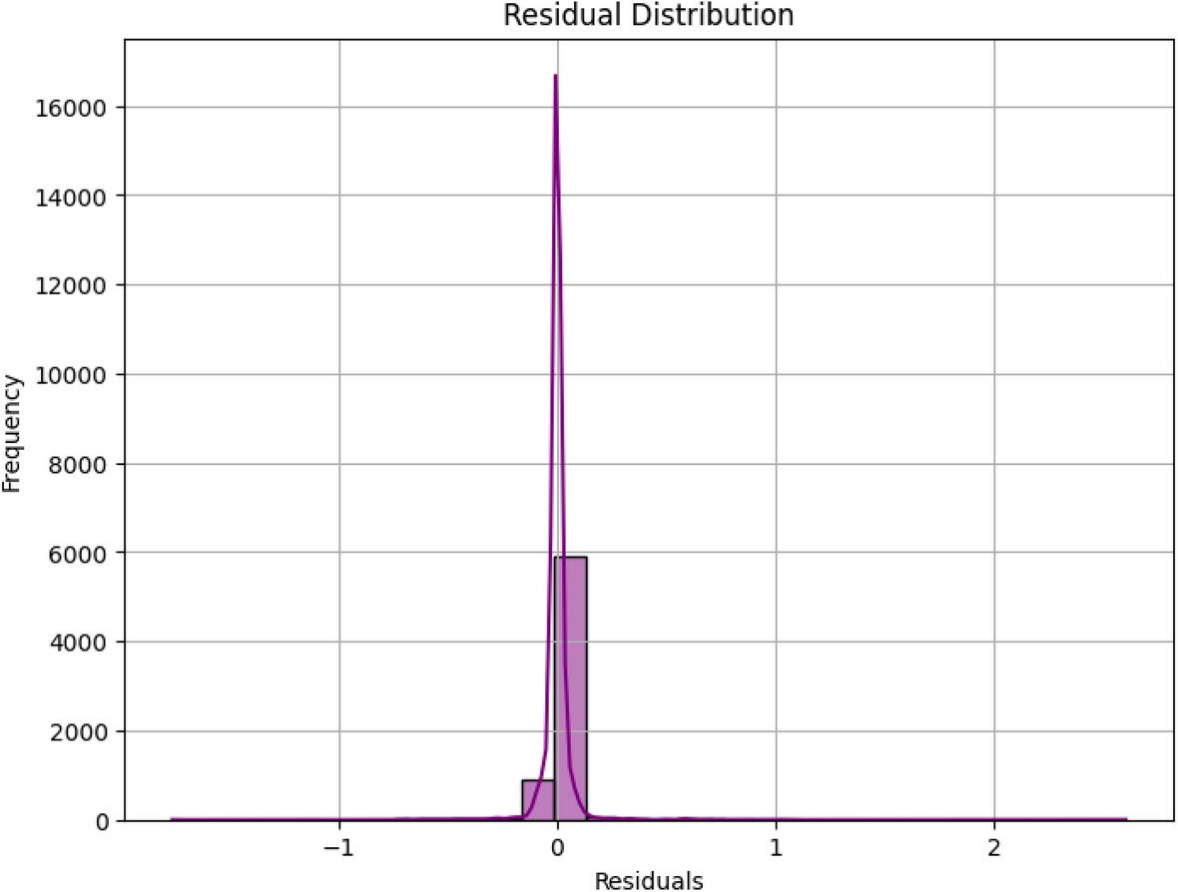
Residual histogram: performance of the MetaStackD model.

**Fig. 16:**
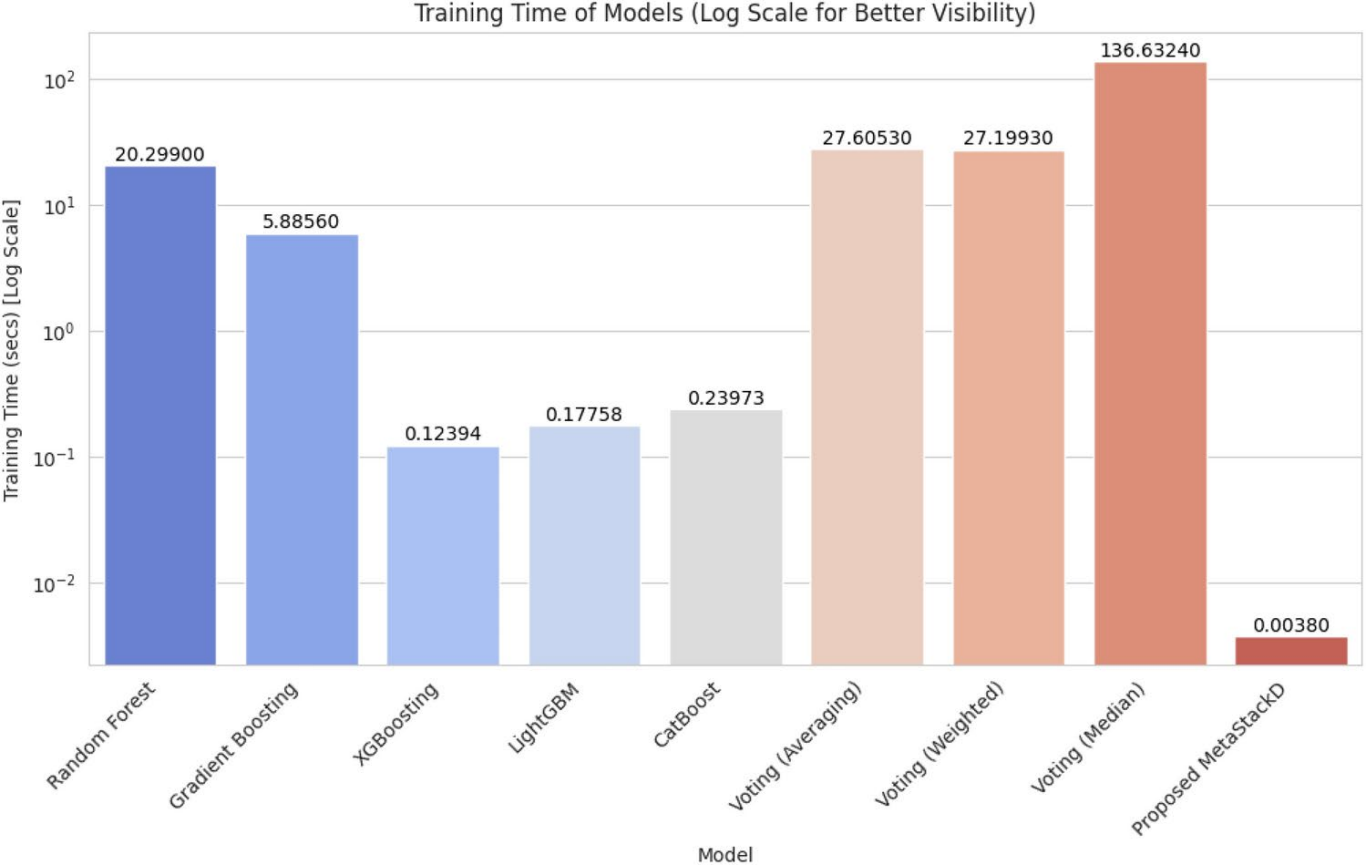
Comparitive analysis of training time across ALL base models, voting ensembles and proposed MetaStackD.

**Fig. 17:**
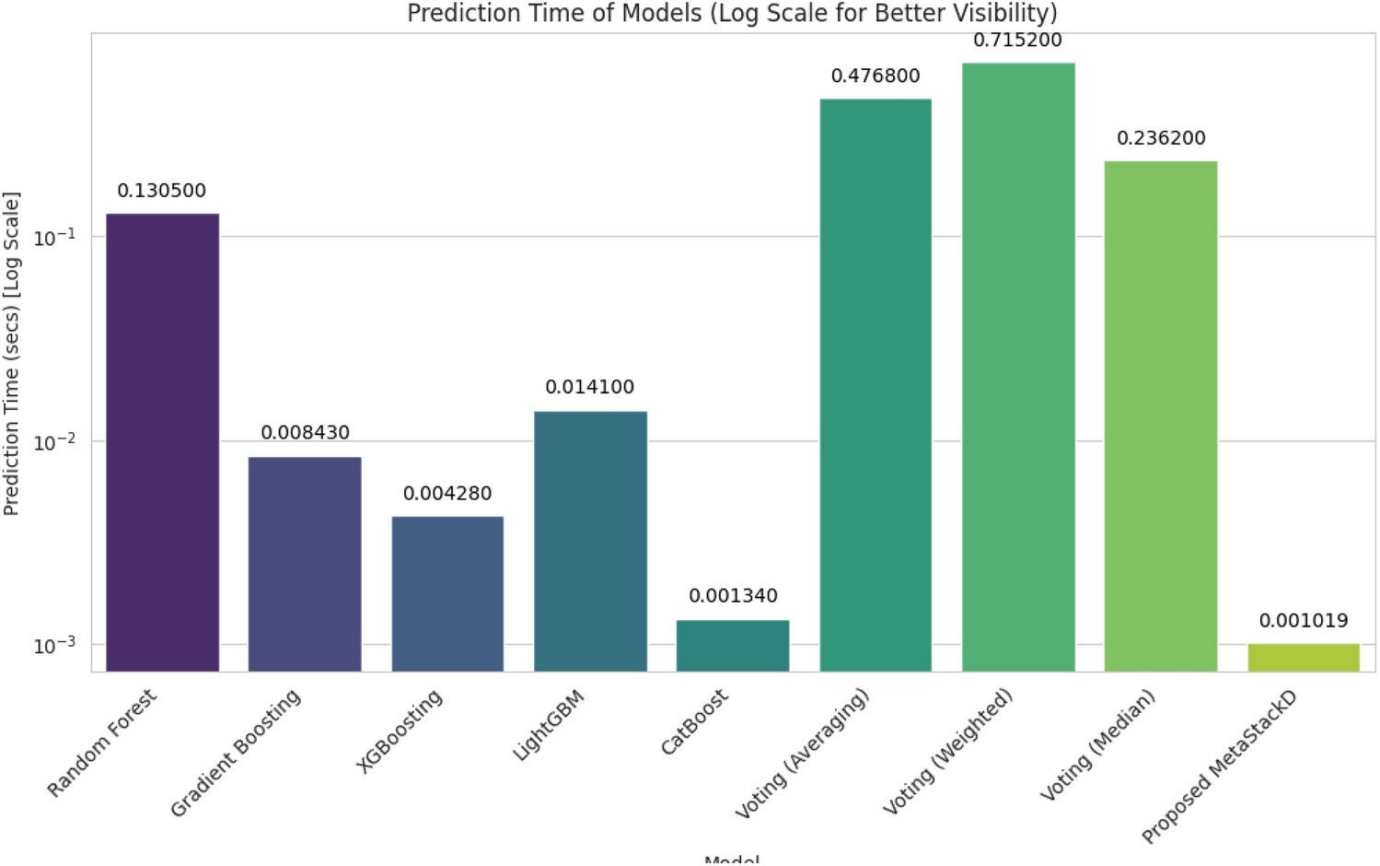
Comparitive analysis of prediction time across ALL base models, voting ensembles and proposed MetaStackD.

- The ensemble approach proposed outperforms the traditional standalone regression models, which is evident from the rate of the prediction error represented by the MSE.
- Over-fitting issues are handled by the proposed framework, as the bias and variance are well-balanced.
- Since various regression models are used as base learners, and the meta-features are used to build a new ensemble model, the unique strengths of each model are leveraged to land with a more robust model for handling diverse data sets.
- The variance and bias errors are reduced when the proposed methodology is used, which is depicted by the residual histogram in Fig. 15. The residual histogram is symmetric and centred around 0. This indicates that the model’s predictions are unbiased and that the errors are equally distributed above and below the actual values. As shown in Fig. 15, the distribution is highly peaked at 0. This means that the predictions given by the proposed MetaStackD model are very close to the actual values. The slight tail seen towards the right illustrates that the model overpredicts in some instances.
- The training time required for training the data set using the proposed MetaSTackD model is lesser when compared to all standalone base models as well as the three voting-based ensemble models as depicted in Fig. 16. The reason behind this is that the meta-model utilizes only the meta-features learnt by the base learners and uses these for learning. Hence, the feature space is still reduced which further reduces the training time.
- The prediction time required is also less when compared to the other models as the meta-model is computationally less expensive and more efficient, as shown in Fig. 17.
- The model is also best suited for multiple data sets with different patterns of data as the base learners used are well equipped to learn high variance, noisy and non-linear relationships. Hence, they do not exhibit any scalability issues in real time.
- Since the meta-model uses only the meta-features from the base learners, the size of the model is only 5.3 MB, which is lesser when compared to all the models. The deployment of this MetaStackD model is easier in real time and can be utilized for any IoE environment. The sensors and the controllers in the smart applications are usually limited in size, and hence, the size of the model plays a major role in deployment.

From all the experimental analysis done using the standalone base learners, voting-based ensembles and the proposed MetaStackD, we can conclude that the proposed MetaStackD is the best model as it has the lowest MAE, MSE and RMSE among all models. Also, it shows the highest $R^2$ value, which specifies that the variance in data is almost captured and explained. Also, the proposed model is strong in capturing complex relationships. All the performance measures, computational measures, feature importance analysis and sensitivity analysis clearly show that the proposed MetaStackD is robust enough to be deployed in a real-time environment. This can also help in improving the functioning of the smart devices in the IoE environment.

### Challenges, limitations and future enhancements

From the model evaluations performed and the experiments conducted, the challenges, limitations and future enhancements are discussed in this section. Among all the models, Median-based voting model and random forest have high training time, which makes them impractical for large-scale applications. Though they show higher accuracy and less error rate, these models consume significant memory thereby limiting scalability. XGBoost and CatBoost are faster and have excellent prediction speed but the accuracy is not as expected. Though MetaStackD has excellent prediction speed, low training time, low prediction time, less computational cost and smaller deployment size, the performance of the model on unseen data needs to be tested for generalization. For further optimizing the computational efficiency, model pruning can be implemented in future. Feature selection or quantization can still reduce memory usage. Experiments need to be conducted to accelerate the model training and inference using Federated Learning concepts. The performance of the proposed MetaStackD can be enhanced with additional base learners. In future, neural network stacking needs to be investigated.

## Conclusion

This research work proposes an algorithm RFRImpute for filling the missing values in the real-time data set obtained from the Chicago Park District IoT device, as there are non-linear relationships among the features. The framework MetaStackD is proposed using base models: Random Forest, Gradient Boosting, Extreme Gradient Boosting, Light Gradient Boosting Machine, and Categorical Boosting. This framework can be utilized in real-time for predicting the RBL of any sensor-based device deployed in the IoE environment across multidisciplinary domains. The prediction accuracy is increased by 1.4% when compared with the weighted and median voting regressor-based ensemble approaches, 5.3% when compared to XGBoost and LightGBM, 11.3% when compared with Gradient Boosting and 13.2% when compared with CatBoost. Though there is no difference in prediction accuracy when a standalone random forest regressor is used, the training time required is reduced from 20.29 to 0.004 s, and the prediction time is reduced from 0.13 to 0.001 s when the proposed framework is used. In future, the focus can be on utilizing multiple real-time data sets to leverage the functioning of the proposed framework.

## Data Availability

The datasets used and/or analysed during the current study are available from the corresponding author upon reasonable request.
